# The *NAC17–PUB30* module enhances drought stress tolerance by regulating adventitious root development in apple

**DOI:** 10.1093/plphys/kiag488

**Published:** 2026-07-10

**Authors:** Bingyang Du, Xiang Zhang, Yuqin Xiao, Maihemuti Turupu, Zhengyang Wen, Xinyu Wang, Shuo Wang, Tingting Sun, Tianhong Li

**Affiliations:** Frontiers Science Center for Molecular Design Breeding, College of Horticulture, China Agricultural University, No. 2 Yuanmingyuan West Road, Haidian District, Beijing 100193, China; Frontiers Science Center for Molecular Design Breeding, College of Horticulture, China Agricultural University, No. 2 Yuanmingyuan West Road, Haidian District, Beijing 100193, China; Frontiers Science Center for Molecular Design Breeding, College of Horticulture, China Agricultural University, No. 2 Yuanmingyuan West Road, Haidian District, Beijing 100193, China; Frontiers Science Center for Molecular Design Breeding, College of Horticulture, China Agricultural University, No. 2 Yuanmingyuan West Road, Haidian District, Beijing 100193, China; Frontiers Science Center for Molecular Design Breeding, College of Horticulture, China Agricultural University, No. 2 Yuanmingyuan West Road, Haidian District, Beijing 100193, China; Frontiers Science Center for Molecular Design Breeding, College of Horticulture, China Agricultural University, No. 2 Yuanmingyuan West Road, Haidian District, Beijing 100193, China; Frontiers Science Center for Molecular Design Breeding, College of Horticulture, China Agricultural University, No. 2 Yuanmingyuan West Road, Haidian District, Beijing 100193, China; Frontiers Science Center for Molecular Design Breeding, College of Horticulture, China Agricultural University, No. 2 Yuanmingyuan West Road, Haidian District, Beijing 100193, China; Frontiers Science Center for Molecular Design Breeding, College of Horticulture, China Agricultural University, No. 2 Yuanmingyuan West Road, Haidian District, Beijing 100193, China

## Abstract

Rootstocks critically influence apple growth, development, and stress tolerance, a process likely mediated by key transcriptional regulators. Among these, NAC transcription factors are pivotal for stress responses, yet their roles in the differential drought resistance of apple rootstock varieties remain unclear. This study identified NAC transcription factor 17 (*NAC17*), which exhibits contrasting drought-responsive expression: it is strongly induced in drought-resistant wild apple (*Malus sieversii*) but remains unresponsive in drought-sensitive M26 (*Malus domestica*). Functional analysis demonstrated that MsNAC17 acts as a positive regulator of drought tolerance. Transgenic M26 plants overexpressing *MsNAC17* showed enhanced drought resistance and promoted adventitious root development, whereas silencing *MsNAC17* increased drought susceptibility. MsNAC17 directly activated the expression of LATERAL ORGAN BOUNDARIES DOMAIN 64 (*MdLBD64*), a key root development regulator, and *MdYUCCA11*, a crucial auxin biosynthesis gene. Furthermore, the E3 ubiquitin ligase Plant U-box 30 (MdPUB30) negatively regulated MsNAC17 protein stability by promoting its degradation via the ubiquitin-proteasome pathway. The differential drought responses between rootstocks were attributed to distinct upstream regulation: in *M. sieversii*, the transcription factor MYB47 activated *MsNAC17* expression, whereas in M26, bZIP46 upregulated *MdPUB30* under drought stress. These results establish the *NAC17-PUB30* regulatory module as a central hub controlling drought tolerance by coordinating root architecture and auxin homeostasis, providing molecular targets for breeding drought-resistant apple rootstocks.

## Teaser

A regulatory circuit that boosts root growth and auxin production in apple trees enhances drought tolerance, offering a target for breeding hardier dwarf rootstocks.

## Introduction

Apple plants (*Malus domestica*) are frequently exposed to drought stress, which can significantly restrict their growth, development, and productivity (Zhu et al. 2024). Drought-resistant rootstocks are routinely used in commercial apple production and enhance the scion's tolerance to drought stress by improving water and nutrient uptake, regulating phytohormone balance, and activating antioxidant defenses (Valverdi et al. 2019). By contrast, drought-sensitive rootstocks result in stunted growth and metabolic disruptions in the scion under drought-stress conditions (Lordan et al. 2017). The drought tolerance of rootstocks is closely related to the development of their root systems.

A key genetic resource for drought tolerance is wild apple (*Malus sieversii*), a wild apple species known for its vigorous growth, extensive root system, and strong drought resistance (Duan et al. 2017). *MsDREB6.2* is a critical gene in the drought response of *M. sieversii*: its overexpression enhanced root development and stress tolerance, while its suppression reduced drought resilience (Liao et al. 2017). Additionally, the promoter region of *MsGH3.5* from *M. sieversii* contains numerous hormone-responsive and abiotic stress responsive cis-elements (Yuan et al. 2013). Overexpression of *MsGH3.5* enhances auxin degradation and cytokinin biosynthesis in transgenic apple lines, thereby influencing plant growth, development, and architecture (Zhao et al. 2020). Selecting drought-resistant rootstocks for apple cultivation in regions with limited water availability enhances water-use efficiency, promotes water conservation, and improves fruit yield and quality (Xiong et al. 2006; Heinitz et al. 2015). However, dwarfing rootstocks, including M26, are widely used in apple production due to their higher fruit-bearing capacity and ease of management. The shallow and underdeveloped root systems of dwarfing rootstocks result in poor drought resistance, limiting their application range (Feng et al. 2023). Consequently, employing modern biotechnological approaches to breed drought-tolerant dwarf apple rootstocks has become an important focus of research given the potential to greatly expand apple cultivation regions while synergistically boosting productivity and fruit quality.

Numerous TFs participate in drought-stress responses in plants by regulating root system development. Knockout of the HD-Zip TF gene *Gmhdz4* enhanced drought tolerance by promoting root development in soybean (*Glycine max*) (Zhong et al. 2022). Heterologous expression of the maize (*Zea mays*) TF *ZmWRKY79* in Arabidopsis (*Arabidopsis thaliana*) increased the number of lateral roots, thereby improving the survival rate under drought conditions (Gulzar et al. 2021). Furthermore, the ZmbZIP89 TF influences lateral root elongation and drought tolerance in maize by regulating cortex/epidermis-specific gene expression (Li et al. 2025). The OsNAC41–RoLe1–OsAGAP module enhances drought resistance in upland rice (*Oryza sativa*) by regulating root length (Han et al. 2024). In poplar (*Populus deltoides*), the NF–YB21–FUS3–NCED3 pathway regulates root growth under drought stress, while PeFUS3 from Euphrates poplar (*P. euphratica*) coordinates lateral root development and drought resistance by regulating auxin transport and abscisic acid (ABA) signaling pathways (Zhou et al. 2020; Liu et al. 2025a, 2025b). Thus, diverse molecular mechanisms underlie the coordinated regulation of root development and drought tolerance across plant species.

Several NAC TFs are associated with drought tolerance (Ma et al. 2024). OsNAC023 is translocated to the nucleus in rice under stress conditions and enhances both drought resistance and thermotolerance by regulating multiple physiological processes (Chang et al. 2024). OsNAC87 improves drought tolerance by activating the expression of its downstream target gene *OsGSTU37*, which helps maintain reactive oxygen species (ROS) homeostasis (Yu et al. 2024). Furthermore, RcWRKY71 is activated by RcNAC091 and participates in the drought response by positively regulating the ABA signaling pathway in rose (*Rosa chinensis*) (Geng et al. 2023). Overexpressing *MdNAC1* enhanced photosynthesis and ROS-scavenging enzyme activity in apple, thereby improving drought tolerance (Jia et al. 2019). MsNAC022 from *M. sieversii* positively regulates drought tolerance by upregulating genes that encode antioxidant enzymes (Peng et al. 2022). Although extensive research has explored the relationship between NAC TFs and drought response, the regulatory network of NAC TFs that mediates root system development in apple remains to be elucidated.

The ubiquitin-proteasome system (UPS) is another crucial regulatory mechanism in the plant drought response (Kelley 2018). This system, consisting of the 26S proteasome, ubiquitin, and 3 enzymes (E1, E2, and E3), tags and degrades target proteins (Xu and Xue 2019). E3 ubiquitin ligases, particularly those in the Plant U-box (PUB) family, are crucial for determining substrate specificity and modulating drought responses. For example, Arabidopsis PUB25/26 enhance cold tolerance by degrading MYB15 (Wang et al. 2019). In the woody plant Populus, PalPUB79 positively regulates ABA-dependent drought tolerance by ubiquitinating PalWRKY77 (Tong et al. 2021). However, some PUB family members function as negative regulators of drought responses, including CaPUB24 in pepper (*Capsicum annuum*), which affects ABA-mediated drought responses (Bae et al. 2024) and GmPUB21 in soybean, which influences stomatal regulation (Yang et al. 2022a, 2022b). IbPUB52 negatively regulates drought tolerance in sweet potato (*Ipomoea batatas*) (Lyu et al. 2024), while U-Box51 positively regulates the drought response in potato (*Solanum tuberosum*) (Wei et al. 2024). Although the roles of PUB family members in mediating plant drought resistance have been extensively studied, the relationships between PUB and NAC TFs, as well as their potential roles in cooperatively regulating plant drought tolerance, remain largely unexplored.

In this study, we reveal the *NAC17-PUB30* regulatory module that determines the divergent drought responses between *M. sieversii* and M26 rootstock. Combining genomic and functional analyses, we show that NAC transcription factor 17 (*NAC17*) mediated drought tolerance operates through adventitious root formation, while Plant U-box 30 (PUB30) fine tunes this pathway via ubiquitination. Our findings provide a molecular basis for improving drought tolerance in dwarfing apple rootstocks.

## Results

### Identification of the drought-responsive NAC17 gene

**Figure 1:**
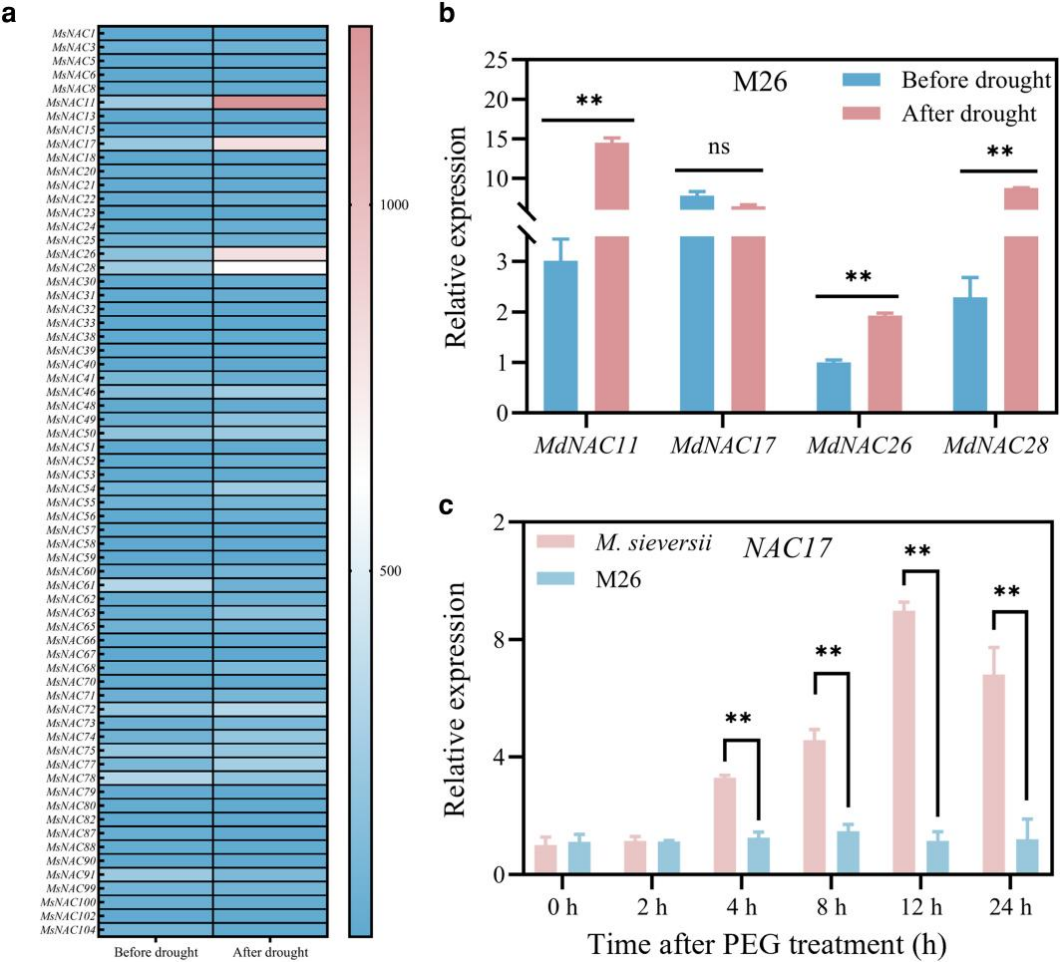
Changes in *NAC* expression in *M. sieversii* and M26 under drought stress. a) Heatmap showing relative *NAC* gene expression levels in *M. sieversii* under drought stress, as detected by RT-qPCR. The heatmap color scale indicates relative expression levels (color key shown in the figure). b) Expression levels of various *NAC* genes in M26 under drought stress, as detected by RT-qPCR. Relative expression levels were normalized to the expression level of *NAC26* in untreated plants (before treatment, set as 1). Drought stress was induced using 20% PEG6000 treatment for 14 d. At the start of treatment, plants were 6 wk old after transplanting into soil. Adventitious root tissues from M26 were used for analysis. c) Relative *NAC17* expression levels in *M. sieversii* and M26 across time points under drought stress were detected by RT-qPCR. Relative expression levels were normalized to the value at 0 h in *M. sieversii* (set as 1). Drought stress was induced using 20% PEG6000 treatment. Adventitious root tissues from *M. sieversii* and M26 were used for analysis in panels a), b), and c) as indicated: a) *M. sieversii* only, b) M26 only, and c) both. Error bars represent the mean ± SE (*n* = 3); ns, no significant difference. **P < 0.01 (two-way ANOVA with Tukey's multiple comparisons test).

To investigate the roles of MsNAC family members in the drought resistance of apple rootstocks, we conducted genome-wide identification and bioinformatics analysis using the drought-resistant rootstock *M*. *sieversii* and identified 104 *NAC* family members. Based on RT-qPCR expression profiling, we identified 4 *NAC* genes (*MsNAC11*, *MsNAC17*, *MsNAC26*, and *MsNAC28*) from *M. sieversii* roots that exhibited ≥3-fold changes in expression under drought stress (Fig. 1a). Among these, *NAC17* exhibited significant drought-induced expression in *M. sieversii* but showed a minimal response in the drought-sensitive dwarfing rootstock M26 (Fig. 1a and b). After 12 h of drought treatment, *NAC17* expression was approximately 5 times higher in *M. sieversii* than in M26 (Fig. 1c), possibly explaining the different levels of drought resistance between these rootstocks. A phylogenetic analysis identified MsNAC48, MsNAC64, and MsNAC99 as the primary homologs of MsNAC17 (Figure S1a). However, expression analysis revealed that *MsNAC64* transcripts were undetectable under both normal and drought conditions. Although *MsNAC48* and *MsNAC99* were expressed, their transcript abundance was constitutively low compared to that of *MsNAC17*, and neither gene was induced under drought stress (Figure S1b). Therefore, based on its dominant and highly inducible expression profile, MsNAC17 was prioritized for subsequent functional characterization.

### Heterologous expression MsNAC17 enhances drought resistance

**Figure 2:**
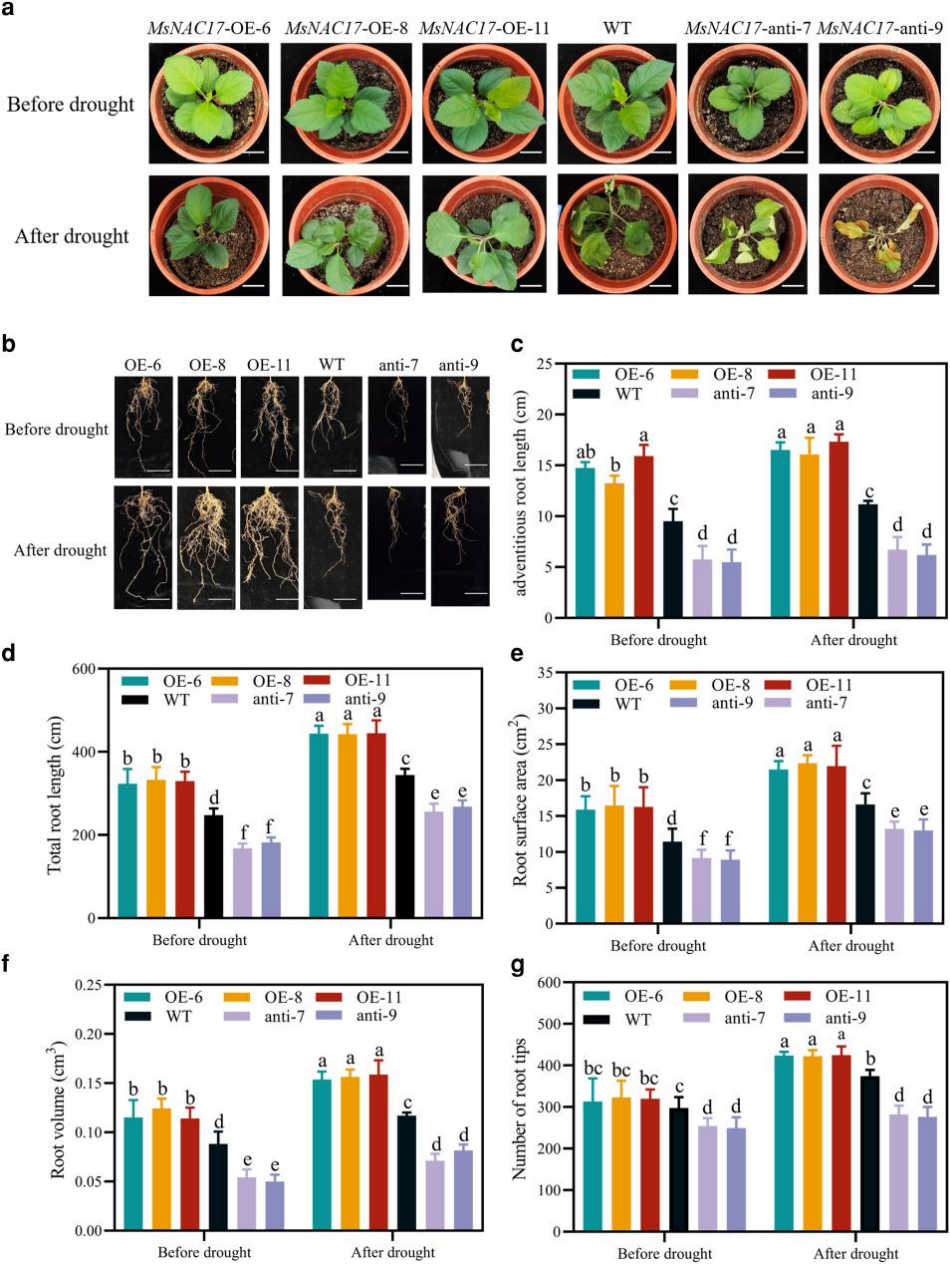
*MsNAC17* transgenic plants show altered drought resistance and root development in the M26 background. a) Phenotypic comparison of aerial parts among *MsNAC17*-overexpressing, silenced, and wild-type plants under drought stress (scale bar, 2 cm). b) Belowground phenotypic differences between *MsNAC17* transgenic and wild-type plants under drought treatment (scale bar, 3 cm). c to g) Statistical analysis of total adventitious root length per plant, total root length per plant, root surface area per plant, total root volume per plant, and root tip number per plant in various genotypes subjected to drought stress (ie, all roots added together for a single plant). Error bars represent the mean ± SE (*n* = 10), with different letters indicating significant differences (*P* < 0.05, one-way ANOVA with Tukey's HSD test). OE-6, OE-8, and OE-11 denote 3 independent *MsNAC17* overexpressing lines in the M26 background, while anti-7 and anti-9 represent 2 *MsNAC17*-silenced lines. Plants were subjected to 14 d of natural drought treatment under field conditions. Abbreviation: WT, wild type.

To further investigate the functional characteristics of MsNAC17, we transiently expressed the MsNAC17-GFP fusion protein in tobacco (*Nicotiana benthamiana*) epidermal cells. Unlike the GFP control, MsNAC17-GFP specifically localized to the nucleus (Supplemental Figure S2a), indicating its role as a nuclear TF. Amino acid sequence alignment using DNAMAN 9.0 revealed that NAC17 contains 5 subdomains (I−V), with subdomains III and IV being highly conserved (Figure S2b). These domains likely participate in DNA binding and transcriptional regulation. PCR amplification revealed that the coding sequences of *NAC17* were identical between *M. sieversii* and the M26 rootstock. Therefore, we cloned *MsNAC17* from *M. sieversii* and conducted both overexpression and silencing experiments in the M26 rootstock. Successful transformation was confirmed at both the transcriptional and DNA levels (Figure S3). Under normal growth conditions, no phenotypic differences were observed among overexpression lines, silenced lines, and wild-type plants. However, after 14 d of natural drought stress, the overexpression lines exhibited enhanced drought tolerance with less leaf curling and wilting, while the silenced lines showed severe damage, although there were no significant differences in plant height (Fig. 2a). Soil water content monitoring showed that soil moisture declined from approximately 76.5% to 25.2% in all genotypes during the drought period, with no significant differences among them (Figure S4). This confirms that the phenotypic differences resulted from distinct physiological responses rather than variation in drought severity. Under drought stress, the overexpression lines accumulated approximately 41.32% more proline than wild-type plants. Three overexpression lines showed reductions in malondialdehyde (MDA) contents of 25.18%, 37.25%, and 31.25%, respectively. The silenced lines displayed opposite trends in terms of both proline and MDA levels (Figure S5). Statistical analysis of the survival rates among the transgenic plant lines revealed that, during natural drought treatment, the survival rate of *MsNAC17* overexpression lines was significantly higher than that of wild-type plants and silenced *MsNAC17* lines. Under 14 d drought stress, the survival rate of *MsNAC17* overexpressing plants was on average 47.14% higher than that of wild-type plants and 79.12% higher than that of *MsNAC17* silenced plants (Figure S6).

*MsNAC17* overexpression lines exhibited an increase in adventitious root length under both normal and drought conditions compared to the wild type, while the silenced lines had a reduction in root length (Fig. 2b and c). To precisely quantify root growth under drought stress, we calculated the percent increase in root length over the 14 d treatment period. The mean adventitious root length of the 3 overexpression lines increased by 31.99%, significantly higher than the 17.54% increase in wild-type M26. The 2 silenced lines showed only a 10.43% increase (Fig. 2b and c). These results indicate that MsNAC17 significantly enhances the root growth capacity of apple rootstocks under drought stress, and the growth advantage conferred by its overexpression constitutes an important physiological basis for improved drought tolerance. Besides, the overexpression lines had significantly greater total root length, root surface area, root volume, and root tip number than both the wild-type and silenced plants (Fig. 2d–g). These results demonstrate that MsNAC17 enhances drought resistance by improving osmotic regulation and reducing membrane lipid peroxidation. Additionally, MsNAC17 positively regulates root development to increase drought tolerance.

Given the central role of oxidative stress in drought damage, we assessed whether *MsNAC17* overexpression improves ROS scavenging capacity, we measured ROS levels and antioxidant enzyme activities. *MsNAC17* overexpression significantly increased antioxidant enzyme activities (SOD, CAT, POD, APX) and reduced ROS accumulation (Figure S7). These findings indicate that MsNAC17 improves drought resistance by promoting root development, enhancing osmotic regulation, and boosting antioxidant capacity.

### MsNAC17 promotes root development by regulating MdLBD64 and MdYUCCA11 expression

**Figure 3:**
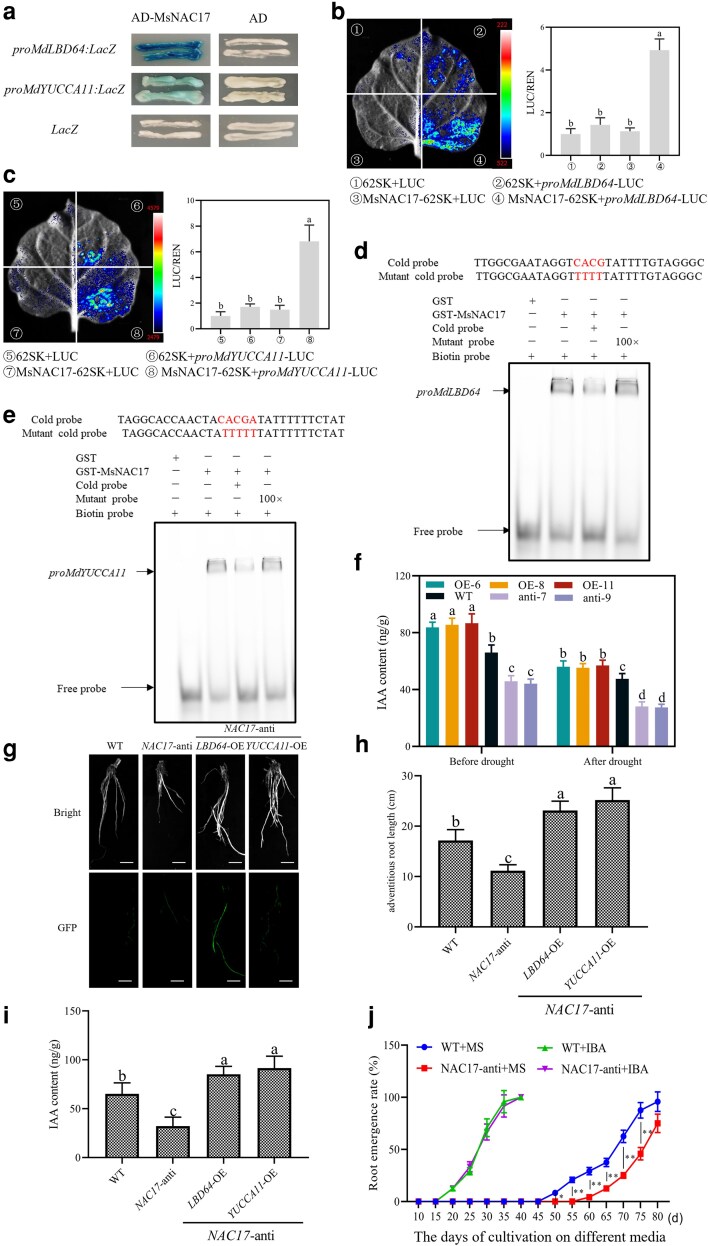
MsNAC17 regulates adventitious root development and auxin biosynthesis. a) Yeast one-hybrid assay showing the binding of MsNAC17 to the promoter regions of *MdLBD64* and *MdYUCCA11* (−2.0Kb). b) MsNAC17 dependent activation of the *MdLBD64* promoter. Left: Representative LUC fluorescence images in *N. benthamiana* leaves. Right: Quantitative LUC/REN ratios. c) MsNAC17 mediated activation of the *MdYUCCA11* promoter. Left: LUC fluorescence images. Right: Normalized LUC/REN activity. d and e) EMSA confirming the binding of MsNAC17 to the promoter regions of *MdLBD64* and *MdYUCCA11*. − and + indicate the absence and presence of binding, respectively. Highlighted text represents the MsNAC17 binding site in the *MdLBD64* and *MdYUCCA11* promoters in the cold probe and the altered binding site in the mutant probe. f) Auxin content of adventitious root tissues in *MsNAC17*-overexpressing, *MsNAC17*-silenced, and wild-type plants. OE-6, OE-8, and OE-11 denote 3 independent *MsNAC17*-overexpressing lines in the M26 background, while anti-7 and anti-9 represent 2 *MsNAC17*-silenced lines. g and h) Phenotypic (g) and statistical analyses (h) of adventitious root length in transgenic *MsNAC17*-silenced and wild-type plants expressing MdLBD64-GFP and MdYUCCA11-GFP fusion proteins. Scale bar, 1 cm. Adventitious root length was measured after 30 d of cultivation. Values are expressed as mean ± SD (*n* = 15 plants per group). i) Auxin content in the roots of transgenic *MsNAC17*-silenced plants expressing MdLBD64-GFP and MdYUCCA11-GFP fusion proteins. Different lowercase letters indicate significant differences. j) Root initiation time and efficiency in *MsNAC17*-anti and WT plants under auxin-free or IBA-supplemented (0.8 mg/L) conditions. Rooting rates were recorded every 5 d. Asterisks indicate significant differences between WT and *NAC17*-anti plants at the same time point (*P < 0.05, **P < 0.01, Student's *t*-test). f, h, i, j) The mean ± SD (*n* = 20 explants per treatment). Different lowercase letters indicate significant differences (*P* < 0.05, one-way ANOVA with Tukey's HSD test).

LBD (LATERAL ORGAN BOUNDARIES DOMAIN) proteins serve as key regulatory factors for lateral root formation in woody plants, and YUCCA proteins are also intricately associated with root development. Therefore, to elucidate the molecular mechanisms by which MsNAC17 regulates root development, we analyzed the expression profiles of root development-related *MdLBD* and *MdYUCCA* family genes in *MsNAC17*-overexpressing and silenced lines. To identify *MdLBD* genes potentially under the direct transcriptional control of MsNAC17, we selected candidates whose expression was consistently elevated in *MsNAC17*-OE lines and correspondingly suppressed in *MsNAC17* silenced lines. Five *MdLBD* genes and 8 *MdYUCCA* genes were consistently and significantly upregulated in *MsNAC17*-overexpressing lines but downregulated in *MsNAC17*-silenced lines, suggesting they may function as downstream effectors of MsNAC17 (Figures S8 and S9). In a yeast one-hybrid assay, MsNAC17 specifically bound to the promoters of LATERAL ORGAN BOUNDARIES DOMAIN 64 (*MdLBD64*) and *MdYUCCA11*, but not to other *MdLBD* or *MdYUCCA* genes (Fig. 3a, Figure S10).

To verify these interactions, we performed a luciferase reporter assay by co-expressing constructs harboring the 2.0-kb *MdLBD64* promoter (*proMdLBD64*-LUC) or the 2.0-kb *MdYUCCA11* promoter (*proMdYUCCA11*-LUC) with the 35S-driven *MsNAC17* effector in *N. benthamiana* leaves. Compared to the control, co-injection of the *MsNAC17* effector and the reporter significantly increased fluorescence intensity and luciferase activity (Fig. 3b and c). Furthermore, in an electrophoretic mobility shift assay (EMSA), MsNAC17 directly bound to the CACG cis-element in the *MdLBD64* promoter and specifically recognized the CACGA element in the *MdYUCCA11* promoter (Fig. 3d and e). These findings demonstrate that MsNAC17 regulates root development by modulating the expression of *MdLBD64* and *MdYUCCA11*.

Since auxin plays a crucial role in root development and stress responses, we hypothesized that MsNAC17 might regulate drought tolerance through modulating auxin biosynthesis or signaling. Therefore, we quantified auxin levels in the roots of transgenic and wild-type plants under both normal and drought conditions. We measured the auxin content in the roots of *MsNAC17* transgenic plants. The auxin content was significantly higher in *MsNAC17*-overexpressing plants and lower in *MsNAC17*-silenced plants than in wild-type plants (Fig. 3f). To further investigate the roles of *MdLBD64* and *MdYUCCA11* in regulating root development, we performed tissue-specific expression analysis. In both the drought resistant rootstock *M. sieversii* and the dwarfing rootstock M26, *MdLBD64* was expressed at significantly higher levels in root tissues compared to the shoot tissues (Figure S11a). Similar expression patterns were observed for *YUCCA11* (Figure S11b), suggesting that both genes primarily function in root tissues. Subsequently, we overexpressed *MdLBD64-GFP* and *MdYUCCA11-GFP* in the *NAC17*-silenced background. The presence of fluorescence signals confirmed the successful overexpression of both fusion proteins (Fig. 3g). Overexpressing either *LBD64* or *YUCCA11* significantly rescued the delayed root development phenotype in the *NAC17* antisense lines. The roots were approximately 34% longer in these plants than in the wild-type plants (Fig. 3h), suggesting that LBD64 and YUCCA11 play critical roles in regulating root development. To further determine whether *MdLBD64* and *MdYUCCA11* are sufficient to mediate MsNAC17 regulated root development under drought stress, we examined the root phenotypes of *LBD64*-OE/NAC17-anti and *YUCCA11*-OE/*NAC17*-anti lines after 14 d of natural drought treatment. Compared to the *NAC17* silenced lines, both complementation lines exhibited significantly increased total root length, root surface area, root volume, and root tip number under drought conditions, reaching levels comparable to those of *MsNAC17* overexpressing plants (Figure S12). These results suggest that *MdLBD64* and *MdYUCCA11* act as key downstream effectors that phenocopy the MsNAC17 mediated root architectural changes under drought stress. Overexpressing either *LBD64* or *YUCCA11* enhanced free IAA accumulation in root tissues, suggesting that both genes are involved in promoting auxin homeostasis, potentially through regulating biosynthesis or other mechanisms such as modulating auxin transport or conjugation (Fig. 3i). To further elucidate the relationship between auxin and root development, we performed tissue culture experiments. Under auxin-free conditions, *NAC17* silenced lines initiated root formation at 60 d compared to 50 d in the wild-type controls, with significantly lower rooting efficiency. However, upon treatment with the auxin IBA, both genotypes initiated root formation within 20 d, with comparable rooting rates (Fig. 3j), demonstrating that exogenous auxin supplementation effectively compensated for the delayed root development in *NAC17*-silenced lines.

### MYB47 regulates genotype-specific MsNAC17 expression

*NAC17* is known to exhibit distinct drought-induced expression patterns in *M sieversii* and M26. To investigate the molecular basis of this variation, we analyzed the *NAC17* promoter regions in both rootstocks, revealing 2 single-base polymorphisms at positions −1,112 (*p1* region) and −35 (*p2* region) (Figure S13a). We constructed GUS vectors for the *p1* and *p2* regions from both rootstocks and transiently expressed them in *N. benthamiana* leaves. After 4 h of 20% PEG treatment (to induce simulated drought stress), GUS staining and activity assays confirmed that the mutation at −1,112 (*p1* region) is the key site responsible for the differential expression of *NAC17* in these 2 rootstocks (Figure S13b).

**Figure 4:**
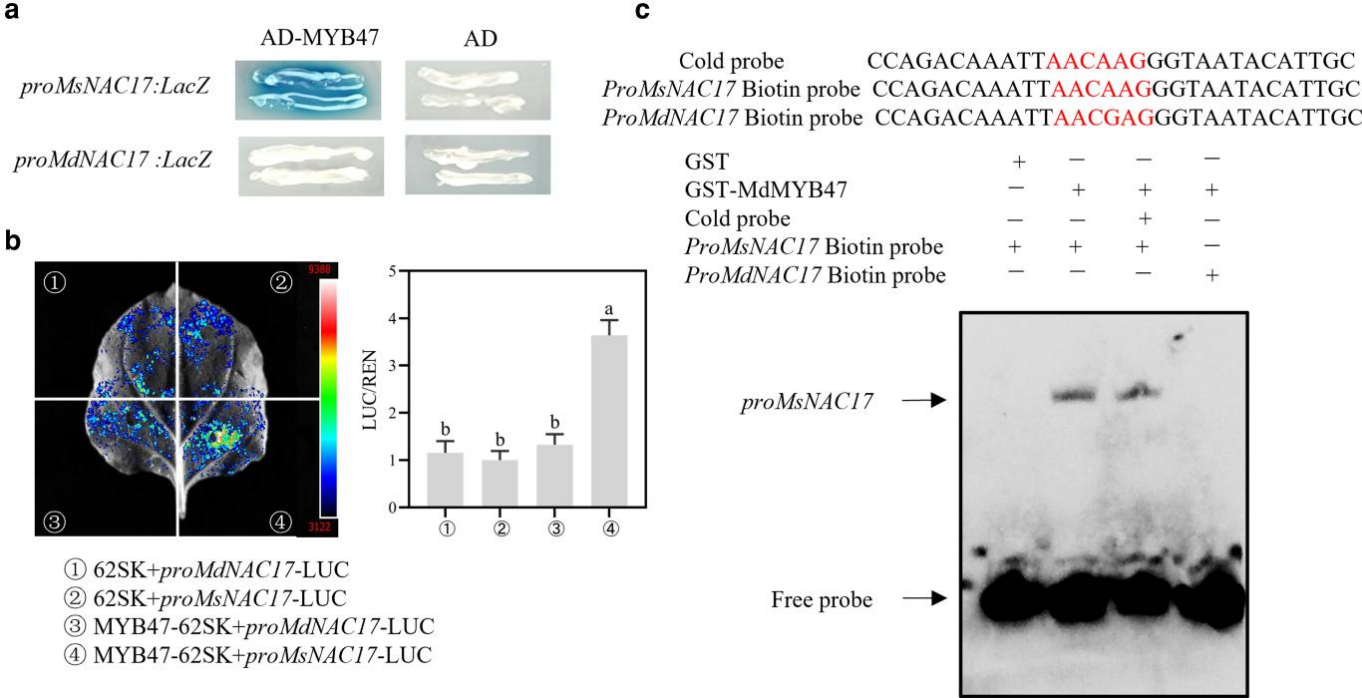
MYB47 regulates *NAC17* expression. a) Yeast one-hybrid assay showing the binding of MYB47 to the *NAC17* promoter region (−2.0 kb) from *M sieversii*. b) Dual-luciferase reporter assay showing LUC fluorescence signals and relative LUC enzyme activities in *N. benthamiana* leaves 48 h after *Agrobacterium* infiltration. 62SK represents the empty vector pGREEN-62-SK, and LUC represents the empty vector pGREEN-0800-LUC. Data are presented as the mean ± SE of 3 biological replicates. Different lowercase letters indicate significant differences. Different letters indicating significant differences (*P* < 0.05, one-way ANOVA with Tukey's HSD test). c) EMSA confirming the binding of MYB47 to the promoter region of *MsNAC17*. − and + indicate the absence and presence of binding, respectively. The highlighted text in the probe represents the MYB47 binding site in the *NAC17* promoter regions of different rootstocks.

Using the *M. sieversii NAC17p1* region as bait, yeast one-hybrid screening identified MYB47 as a TF that specifically binds to the *M. sieversii NAC17* promoter but not to the M26 promoter (Fig. 4a). In dual-luciferase reporter assays, MYB47 significantly enhanced *M. sieversii NAC17* promoter activity but had no effect on the M26 promoter (Fig. 4b). In an EMSA, MYB47 bound to the *M. sieversii NAC17* promoter but not to the M26 sequence (Fig. 4c). *MYB47* expression was upregulated in response to drought stress in both rootstocks, as revealed by RT-qPCR (Figure S14). These findings suggest that MYB47 enhances *NAC17* expression by binding to the *p1* region in its promoter specifically in *M. sieversii*, thereby explaining the differential expression of this gene in the 2 rootstocks. This finding highlights the critical role of the *MYB47-NAC17* module in regulating the differences in drought resistance between *M. sieversii* and M26.

### MdPUB30 mediates the ubiquitination and degradation of MsNAC17

**Figure 5:**
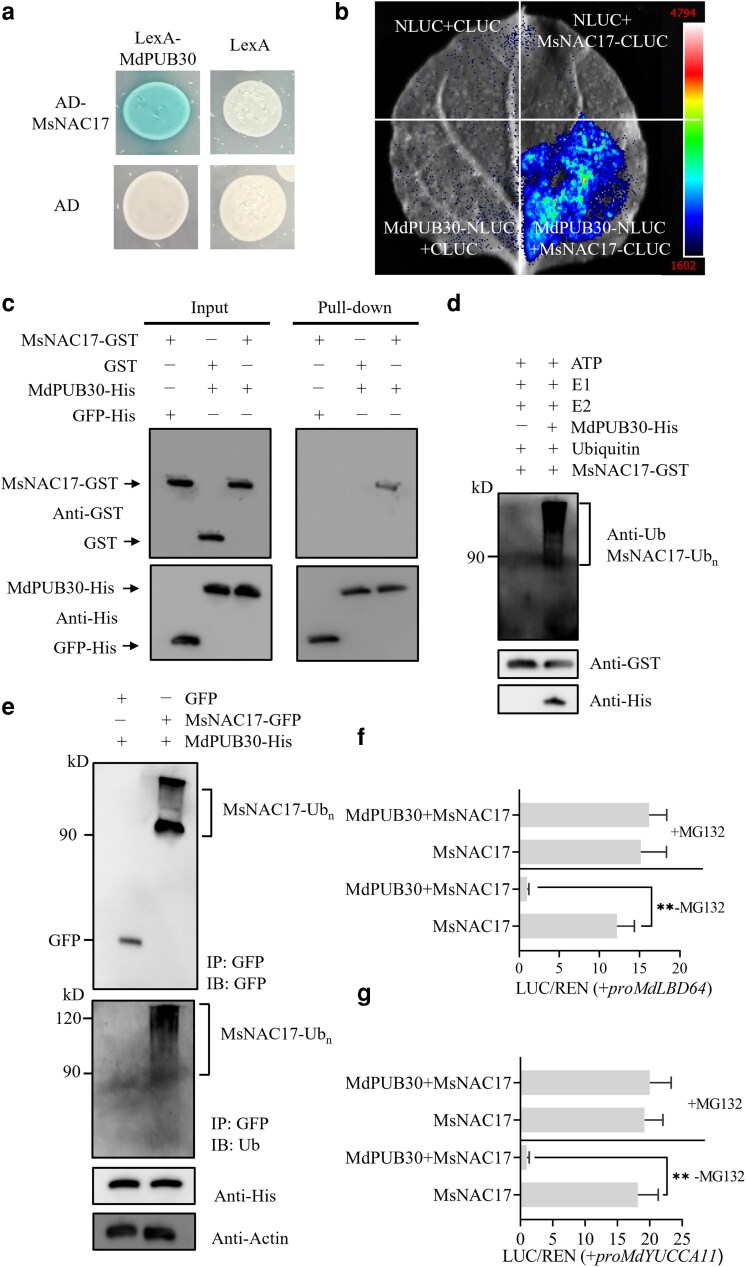
MdPUB30-mediated ubiquitination and degradation of MsNAC17. a) Yeast two-hybrid screening identifying the interaction between MdPUB30 and MsNAC17. Positive yeast cells grew on SD/–Trp/–Leu/–His/X-Gal plates (*n* = 3). b) Luciferase complementation imaging assay confirming the interaction between MdPUB30 and MsNAC17 in *N. benthamiana* leaves. c) Pulldown assay verifying the interaction between MdPUB30 and MsNAC17 in vitro. – and + represent the absence and presence of an interaction, respectively. Anti-GST and anti-His antibodies were used for the analysis. d) In vitro ubiquitination assay demonstrating that MdPUB30 mediates the ubiquitination of MsNAC17. The reaction system included E1 (50 nM), E2 (100 nM), ubiquitin (5 μg), and ATP (2 mM). Immunoblotting detected ubiquitinated bands (indicated by arrows). The negative control lacked MdPUB30. − and + indicate the absence and presence of MdPUB30, respectively. e) Immunoblotting detecting the ubiquitination of MsNAC17-GFP in *N. benthamiana* leaves. *Agrobacterium* containing MsNAC17-GFP and MdPUB30-His was infiltrated into *N. benthamiana* leaves, and samples were collected 48 h later. Total proteins were extracted, immunoprecipitated with anti-GFP antibody (Abcam, 1:5000), and analyzed for ubiquitination using anti-GFP and anti-Ub antibodies. The negative control consisted of leaves transformed only with MsNAC17-GFP. f and g) *N. benthamiana* leaves were transiently infiltrated with *Agrobacterium* containing the MsNAC17, MdPUB30, and effector constructs (*proMdLBD64* and *proMdYUCCA11*). The promoter-LUC reporter assay was performed by detecting LUC fluorescence signals 48 h after infiltration. The experiment was divided into 2 groups: samples with or without MG132 injection. Asterisks indicate significant differences by Student's *t*-test. Error bars represent SD (n = 3 biological replicates), **P < 0.01. Abbreviations: IB, immunoblot, IP, immunoprecipitated.

To elucidate the molecular regulatory network of MsNAC17 in drought resistance, we conducted yeast two-hybrid screening using MsNAC17 as bait and identified the E3 ubiquitin ligase MdPUB30 as an interacting partner (Fig. 5a). Luciferase complementation (LUC) and pulldown assays validated this interaction (Fig. 5b and c). Given the role of MdPUB30 in protein ubiquitination, we examined its ability to mediate the ubiquitination and degradation of MsNAC17 by performing in vitro ubiquitination assays. We detected significant band smearing in the presence of MdPUB30, confirming the ubiquitination-mediated degradation of MsNAC17 (Fig. 5d). To validate this result in plants, we co-infiltrated *N. benthamiana* leaves with constructs encoding MdPUB30-His and MsNAC17-GFP. Immunoblot analysis revealed band smearing in the experimental group, confirming that MdPUB30 mediates the ubiquitination and degradation of MsNAC17 in vivo (Fig. 5e). To identify the critical ubiquitination sites in MsNAC17 targeted by PUB30, we performed a bioinformatic analysis, which predicted Lys56 and Lys173 as potential ubiquitination sites (Table S1). We then generated lysine-to-arginine mutants (K56R, K173R, and the double mutant K56/173R) and co-expressed them with PUB30 in *Nicotiana benthamiana*. The results showed that all mutants accumulated to significantly higher protein levels compared to the wild-type in the presence of PUB30 (Figure S15). This indicates that mutation at Lys56 and Lys173 effectively attenuates PUB30-mediated degradation of MsNAC17, establishing these 2 residues as key sites required for the ubiquitination-dependent turnover of MsNAC17.

Since MsNAC17 positively regulates the expression of *MdLBD64* and *MdYUCCA11*, we examined the effect of MdPUB30 on the regulatory function of MsNAC17. In the *proMdLBD64*-LUC and *proMdYUCCA11*-LUC reporter systems, co-expression of MdPUB30 and MsNAC17 significantly reduced luciferase activity, an effect that was reversed by the proteasome inhibitor MG132 (Fig. 5f and g). These findings indicate that PUB30 reduces the transcriptional activation activity of NAC17 on its downstream target genes (*MdLBD64* and *MdYUCCA11*) by mediating their ubiquitination and degradation.

### PUB30 silencing enhances drought resistance

**Figure 6:**
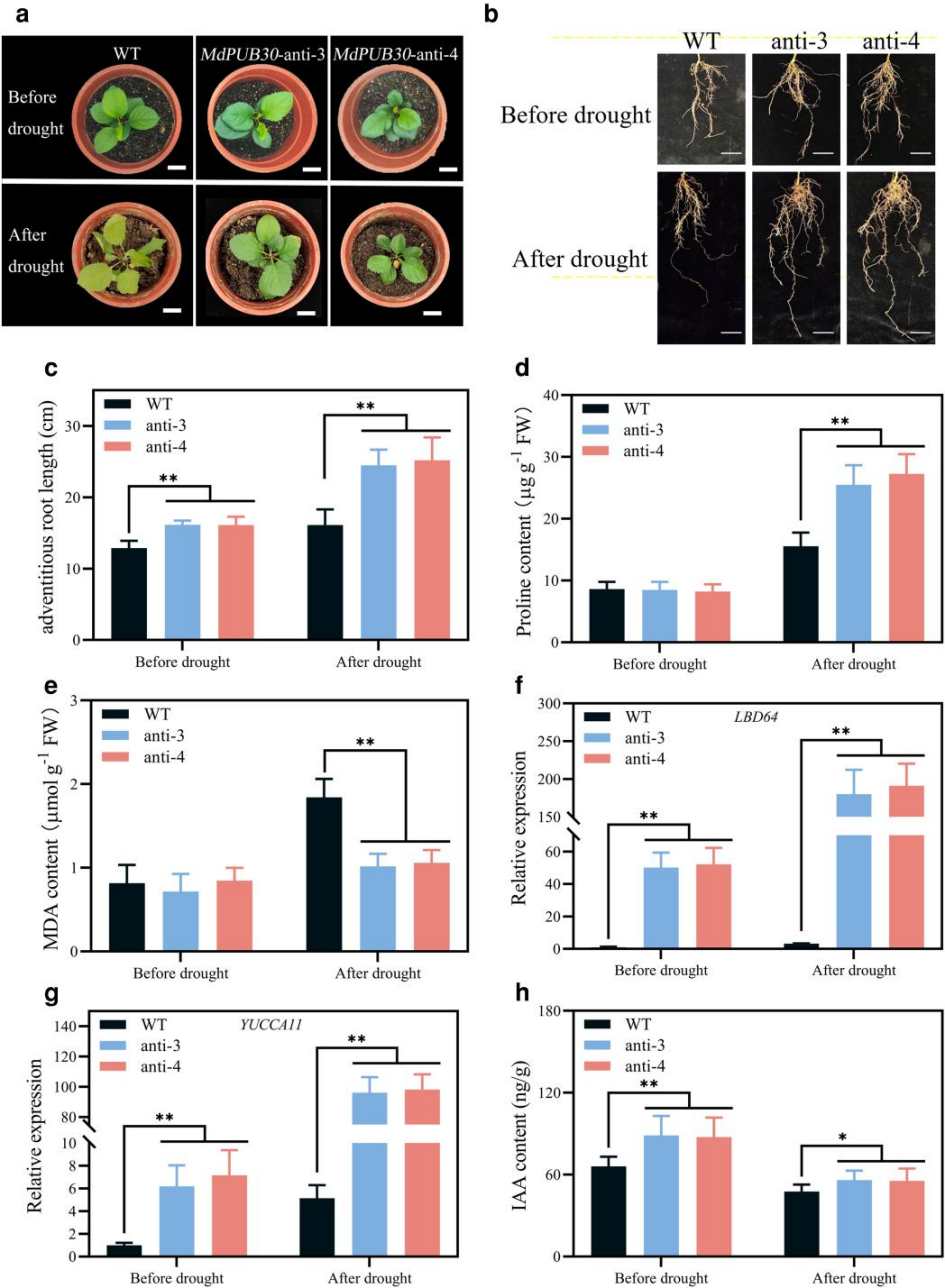
Effects of *MdPUB30* silencing on drought tolerance in M26. a) Aboveground phenotypic comparison between WT and *MdPUB30*-silenced lines. Scale bar, 2 cm. Images were digitally extracted for comparison. b) Underground phenotypic comparison between WT and *MdPUB30*-silenced lines. Scale bar, 2.5 cm. c) Statistical analysis of adventitious root length in WT and *MdPUB30*-silenced lines. d and e) Proline and MDA contents in adventitious root tissues of WT and *MdPUB30*-silenced lines. f and g) Expression of *MdLBD64* and *MdYUCCA11* in adventitious root tissues of WT and *MdPUB30*-silenced lines. h) Auxin content in adventitious root tissues of WT and *MdPUB30*-silenced lines. Error bars represent the mean ± SE (*n* = 3). Asterisks indicate significant differences between WT and silenced plants within the same treatment condition (*P < 0.05, **P < 0.01, Dunnett's test). Absence of asterisks denotes no statistically significant difference. Anti-3 and anti-4 represent 2 *MdPUB30*-silenced lines in the M26 background. Drought treatment was applied for 14 d under natural conditions. *P  *<*  *0.05*, **P  *<*  *0.01*; no label indicates no significant difference. Abbreviations: WT, wild type.

To validate the role of MdPUB30 in drought stress, we constructed an *MdPUB30 antisense* vector to silence *MdPUB30* in M26. Successful transformation and silencing were confirmed at the transcriptional and DNA levels (Figure S16). Under normal growth conditions, *MdPUB30*-silenced and wild-type plants did not exhibit any significant phenotypic differences. However, after 14 d of natural drought treatment, wild-type plants exhibited more severe leaf curling and wilting than *MdPUB30*-silenced plants (Fig. 6a). Soil water content monitoring showed that soil moisture declined from approximately 77.2% to 23.9% in all genotypes during the drought period, with no significant differences among them (Figure S17). This confirms that the phenotypic differences resulted from distinct physiological responses rather than variation in drought severity. Statistical analysis of the survival rates among the transgenic plant lines revealed that, during natural drought treatment, the survival rate of *MdPUB30* silenced lines was significantly higher than that of wild-type plants. Under drought stress, the survival rate of *MdPUB30* silenced plants was on average 48.35% higher than that of wild-type plants (Figure S18). Silencing *MdPUB30* significantly promoted adventitious root development. Under both normal and drought conditions, the root length of *MdPUB30* silenced lines increased by approximately 25.4% and 56.8%, respectively, compared to the wild type (Fig. 6b and c). Furthermore, after 14 d of drought treatment, the average adventitious root length of the 2 silenced lines increased by 50.05%, significantly higher than the 21.27% increase observed in wild-type M26 (Fig. 6b and c). These findings, together with the similar root-promoting effect observed in *MsNAC17* overexpressing plants (Fig. 2), demonstrate that MdPUB30 acts as a negative regulator of root growth, likely by modulating the stability of MsNAC17, thereby reinforcing the role of the *NAC17-PUB30* module in coordinating root development under drought stress. Root scanning analysis revealed that *MdPUB30*-silenced plants had significantly greater total root length, surface area, volume, and tip number than wild-type plants (Figure S19), indicating that silencing MdPUB30 enhances drought resistance by modulating root development.

Under drought stress, *MdPUB30*-silenced plants exhibited significantly higher proline contents and lower MDA contents than the wild-type plants (Fig. 6d and e), suggesting that *MdPUB30* silencing enhances osmotic regulation and mitigates membrane lipid peroxidation by stabilizing MsNAC17 protein levels. Additionally, *MdPUB30*-silenced plants exhibited significantly higher expression levels of *MdLBD64* and *MdYUCCA11*, as well as increased auxin contents in their roots compared to wild-type plants under both normal and drought conditions (Fig. 6f–h). In contrast, in wild-type plants under drought stress, *MdYUCCA11* transcript levels increased while the IAA content decreased (Fig. 6g and h). This apparent inconsistency reflects the complex homeostatic regulation of auxin. Under prolonged drought, enhanced IAA catabolism (eg, oxidation and conjugation) can override the transcriptional upregulation of biosynthesis genes, leading to a net decrease in free IAA. These findings indicate that MdPUB30 negatively modulates drought tolerance and root architecture by promoting the degradation of MsNAC17, thereby attenuating its transcriptional activation of *MdLBD64* and *MdYUCCA11*.

### Mechanism underlying the differential expression of PUB30 in the 2 rootstocks

**Figure 7:**
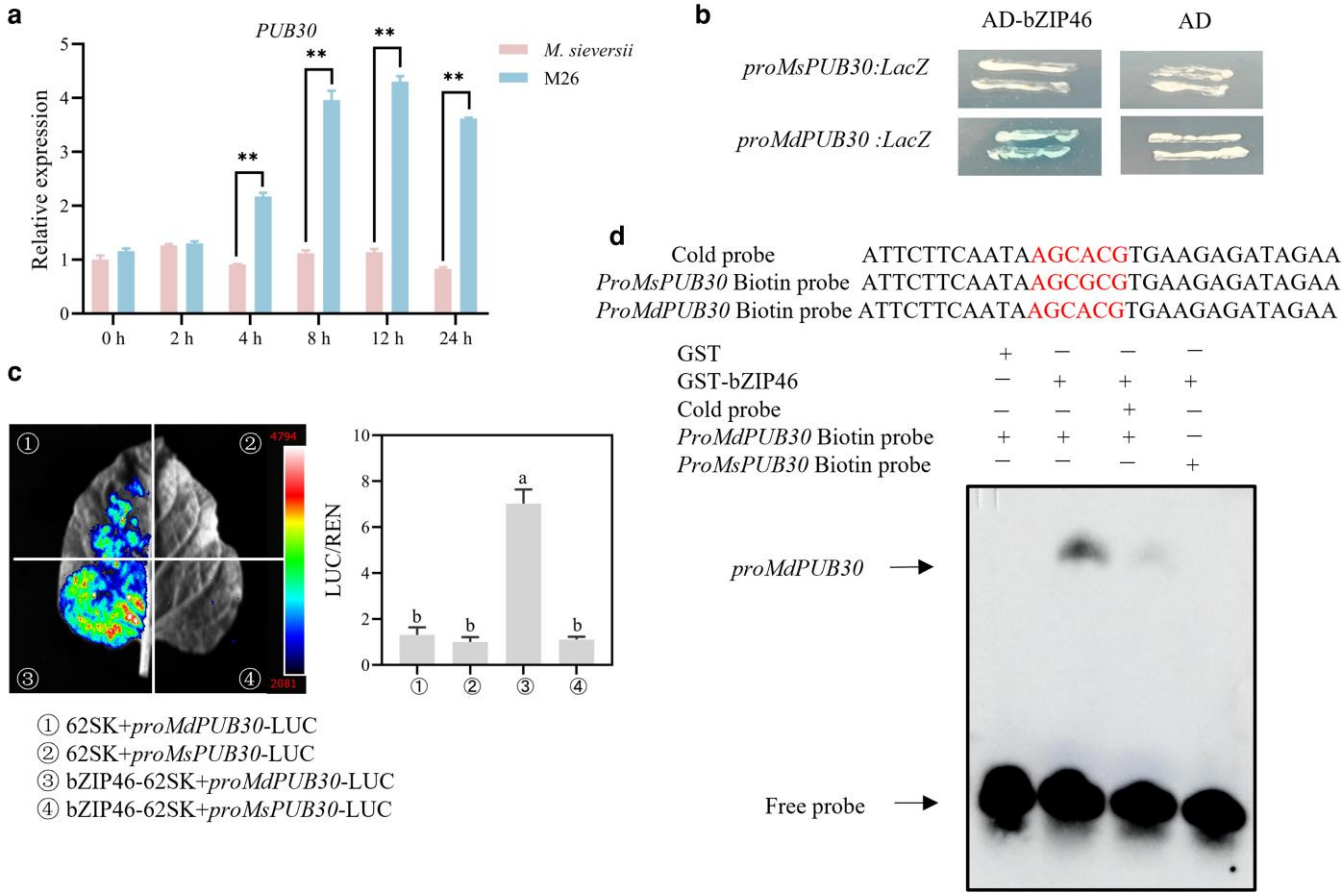
bZIP46 regulates *PUB30* expression. a) RT-qPCR analysis of *PUB30* expression in *M. sieversii* and M26 under drought stress at various time points. Drought stress was applied using 20% PEG6000 in hydroponically grown *M. sieversii* and M26, transplanted from tissue culture and grown hydroponically for 1 month, and samples were collected from the underground parts of plants. Data are presented as the mean ± SE (*n* = 3). **P < 0.01. b) Yeast one-hybrid assay confirming the binding of bZIP46 to the *PUB30* promoter (−2.0 kb) in M26. c) LUC fluorescence signals and relative LUC enzyme activity in *N. benthamiana* leaves 48 h after *Agrobacterium* infiltration measured by in vivo luminescence imaging and a luciferase activity assay, respectively. 62SK represents the empty vector pGREEN-62-SK, and LUC represents the empty vector pGREEN-0800-LUC. Data are presented as the mean ± SE of 3 biological replicates. Different letters indicating significant differences (*P* < 0.05, one-way ANOVA with Tukey's HSD test). d) EMSA confirming the binding of bZIP46 to the *MdPUB30* promoter. − and + represent the absence and presence of binding, respectively. The highlighted text in the probe represents the bZIP46 binding site in the *PUB30* promoter regions of different rootstocks.

To investigate whether *PUB30* responds to drought stress and the molecular basis of its differential expression in *M. sieversii* and M26, we performed RT-qPCR to analyze the drought response patterns of *PUB30* in both rootstocks. *PUB30* expression was significantly induced in M26 under drought stress, with expression levels significantly higher than those in *M. sieversii* between 4 and 24 h of treatment. Conversely, *PUB30* in *M. sieversii* showed almost no response to drought stress (Fig. 7a). The *PUB30* expression pattern inversely correlated with *NAC17* expression in both rootstocks, suggesting that PUB30 regulates drought resistance by modulating NAC17 stability.

To explore the cause of the differential expression of *PUB30*, we analyzed its promoter region. We identified 2 single-base polymorphisms at positions −1,579 (*p1* region) and −840 (*p2* region) (Figure S20a). Using the GUS reporter systems driven by the *p1* and *p2* regions of both rootstocks under 20% PEG-induced drought stress, we identified the polymorphism at −1,579 (*p1* region) as the key determinant of the differential expression of *PUB30* between the 2 rootstocks (Figure S20b).

A yeast one-hybrid screen using the M26 *PUB30p1* region as bait identified bZIP46 as a TF that specifically binds to the *PUB30* promoter in M26 but not in *M. sieversii* (Fig. 7b). In dual-luciferase reporter assays, bZIP46 significantly enhanced M26 *PUB30* promoter activity but had no effect on the *M. sieversii* promoter (Fig. 7c). In an EMSA, bZIP46 directly bound to the M26 *PUB30* promoter but not to the mutated sequence in *M. sieversii* (Fig. 7d). RT-qPCR analysis revealed that *bZIP46* expression increased in response to drought stress in both rootstocks (Figure S21), indicating its role as an upstream regulator of the drought response. These findings suggest that bZIP46 mediates the differential expression of *PUB30* between the 2 rootstocks by specifically binding to the *p1* region of its promoter in M26.

## Discussion

### MsNAC17 enhances drought tolerance in apple rootstocks by promoting adventitious root development and auxin accumulation

High frequency, prolonged droughts severely limit the growth and geographical distribution of crops (McGregor et al. 2020; Wu et al. 2020; Xie et al. 2024). Apple propagation normally relies on grafting, ie, combining vigorous rootstocks with high-quality scions, to enhance plant resilience, yield, and fruit quality. Dwarfing and high-density planting are the predominant cultivation models for modern apple production. Dwarfing rootstocks such as M9 and M26 are widely used in apple production; however, they exhibit weak drought resistance, making them prone to drought stress. Therefore, developing drought-resistant dwarfing rootstocks with broad adaptability is essential for improving production efficiency in major apple-producing regions in China and ensuring the sustainable development of the apple industry.

M. *sieversii* Roem., a wild apple species found in mountainous regions of western China and the Tianshan Mountains of Central Asia (Cornille et al. 2014), exhibits the strongest drought resistance among apple rootstocks (Li and Li, 2005). Furthermore, as a major contributor to the modern cultivated apple genome (Duan et al. 2017), *M. sieversii* serves as a key model for exploring the regulation of the drought-stress response in apple. In recent years, transgenic technology has played an important role in improving plant resistance. Genetic engineering targeting TFs has emerged as a powerful approach for enhancing plant stress tolerance, as drought-responsive TFs significantly improve plant drought tolerance (Yang et al. 2024; Liu et al. 2025a, 2025b). In this study, we identified 104 NAC family member genes in *M. sieversii*, among which 4 (*NAC11*, *NAC17*, *NAC26*, and *NAC28*) were drought-induced (Fig. 1a and b). Of these 4, *NAC17* exhibited the most significant differential expression between drought-resistant (*M. sieversii*) and drought-sensitive (M26) rootstocks (Fig. 1c). Similar differential expression patterns of TF genes across plant varieties have been reported in other crops. For example, in potato, the expression of Dof-TF genes varies significantly between drought-tolerant (Long10) and drought-sensitive (DXY) cultivars (Jin et al. 2024). Similarly, in maize, the expression of HSF B-class genes under salt stress is significantly higher in salt-tolerant than in salt-sensitive varieties, potentially contributing to their differential salt tolerance (Du et al. 2017). The differential expression of *NAC17* between the 2 apple rootstocks identified in this study directly correlates with their distinct levels of drought resistance.

NAC TFs are crucial for plant drought resistance. For instance, SlNAC6 regulates development, drought-stress responses, and fruit ripening in tomato (*Solanum lycopersicum*) (Jian et al. 2021). GmNAC12 positively regulates drought tolerance in soybean (Yang et al. 2022a, 2022b). NtNAC053 confers drought and salt tolerance in tobacco (*Nicotiana tabacum*) by activating downstream stress-responsive genes and antioxidant systems (Li et al. 2022). Additionally, TaNAC5D-2 enhances drought tolerance in wheat (*Triticum aestivum*) by regulating water loss (Ma et al. 2022). Despite extensive research on the roles of NAC TFs in plant adaptation to drought stress, the role of NAC17 in apples has not been characterized.

In this study, overexpressing *MsNAC17* in M26 significantly improved drought tolerance, as evidenced by reduced leaf wilting, enhanced proline accumulation, and lower MDA levels (Fig. 2, Figure S5). We also demonstrated that MsNAC17 influences root architecture by increasing total root length, root surface area, and root tip number, which likely enhances water uptake under drought conditions. This finding extends the known functions of NAC TFs beyond transcriptional regulation to include developmental adaptation. We also showed that MsNAC17 directly activates the expression of *MdLBD64* and *MdYUCCA11*, which encode key regulators of lateral root formation and auxin biosynthesis, respectively (Fig. 3). The functional importance of these targets was evidenced by the rescue of the root developmental and auxin-deficient phenotypes in *MsNAC17* silenced lines upon overexpression of either *MdLBD64* or *MdYUCCA1*1 (Fig. 3). Together, these findings position *MdLBD64* and *MdYUCCA11* as crucial downstream effectors through which MsNAC17 modulates root architecture. The functional importance of these targets was further supported by complementation assays under drought stress conditions. Overexpression of either *MdLBD64* or *MdYUCCA11* in the NAC17 silenced background significantly rescued all key root architectural parameters, including adventitious root length, total root length, root surface area, root volume, and root tip number to levels comparable to those of *NAC17* overexpressing lines (Figure. 12), confirming that these 2 genes are sufficient to phenocopy the NAC17 mediated root architectural changes under drought stress. These findings solidify the role of *MdLBD64* and *MdYUCCA11* as crucial downstream effectors through which MsNAC17 modulates root architecture and drought tolerance. It is noteworthy that despite the positive regulation of auxin biosynthesis by MsNAC17 via *MdYUCCA11*, the overall auxin content in roots was still lower under drought stress compared to well-watered conditions (Fig. 3f). This reduction is consistent with previous studies showing that water deficit often represses auxin biosynthesis or promotes its degradation at the whole-plant level, which may represent an adaptive strategy to limit shoot growth and conserve resources under stress conditions (Wang et al. 2018; Jing et al. 2023; Liu et al. 2023). Importantly, our data demonstrate that *MsNAC17* overexpressing lines maintained relatively higher auxin levels than wild-type or silenced plants under drought, suggesting that MsNAC17 mitigates the stress induced auxin decline, thereby sustaining root development and drought resilience. These findings provide insight into the role of NAC TFs in drought resistance in fruit trees. Furthermore, MsNAC17 enhances drought tolerance by improving osmotic regulation and increasing antioxidant enzyme activity (Figure S7). Our findings suggest that *MsNAC17* is highly induced by drought in *M. sieversii* and regulates multiple downstream target genes, including those related to osmotic regulation and antioxidant activity, thereby contributing to the drought-stress response in apple rootstocks.

### MdPUB30 negatively regulates drought tolerance in apple rootstocks by ubiquitinating and destabilizing MsNAC17

Post-translational modifications of proteins also play significant roles in plant responses to drought stress (Zhao et al. 2024). The UPS regulates protein ubiquitination and degradation in plants. Among its components, PUB family E3 ubiquitin ligases help determine the substrate specificity of the 26S proteasome system in degrading various target proteins (Sullivan et al. 2003). In *Arabidopsis*, the U-box E3 ubiquitin ligases AtPUB18, AtPUB19, AtPUB22, and AtPUB23 negatively regulate ABA-mediated drought responses (Cho et al. 2008; Liu et al. 2011; Seo et al. 2021). Additionally, AtPUB11 is thought to negatively regulate drought tolerance by targeting LRR1 and the receptor-like protein kinase KIN7 for degradation (Chen et al. 2021). GmPUB6, GmPUB8, and GmPUB21 negatively regulate drought tolerance and are involved in osmotic stress and ABA signaling pathways in soybean (Wang et al. 2016, 2020; Yang et al. 2022a, 2022b). However, the roles of PUB family proteins in drought responses in apple have been unknown. Here we identified MdPUB30 as an MsNAC17-interacting protein through yeast two-hybrid screening. We demonstrated that the E3 ubiquitin ligase MdPUB30 regulates MsNAC17 stability through ubiquitination-mediated degradation (Fig. 5a–e), influencing its transcriptional activation of downstream target genes (Fig. 5f and g). Furthermore, mutations at Lys56 and Lys173 of the MsNAC17 protein effectively attenuated the degradation of MsNAC17 mediated by PUB30, indicating that these 2 lysine residues are critical sites for the ubiquitination-mediated degradation of NAC17 by PUB30 (Figure S15). This discovery highlights the critical role of the UPS in plant drought resistance and reveals the role of the *NAC17-PUB30* regulatory module in drought responses in apple. Our findings offer insight into the link between ubiquitination and plant stress responses. Further research should explore whether PUB30 targets other substrates for ubiquitination during drought stress.

To validate the role of MdPUB30 in drought stress, we constructed a *MdPUB30*-silencing vector and used it to transform M26. Under drought stress, Md*PUB30*-silenced lines exhibited significantly enhanced drought resistance, including a 23% increase in adventitious root length (Fig. 6b and c), improved total root length, surface area, and tip number (Figure S16), increased proline content, and reduced MDA content (Fig. 6d and e). Additionally, *MdLBD64* and *MdYUCCA11* expression levels and auxin content were significantly higher in *MdPUB30*-silenced plants than in wild-type plants (Fig. 6f–h), indicating that MdPUB30 affects downstream target gene expression by regulating NAC17 stability. Although *NAC17* is not drought-inducible in M26, it maintains a high basal expression level relative to other *NAC* genes (Fig. 1), which provides a substrate for post-translational regulation by MdPUB30. This explains the drought sensitive phenotype observed when *NAC17* is silenced and the enhanced tolerance when *MdPUB30* is knocked down. However, whether MdPUB30 influences drought-stress responses by ubiquitinating and degrading other proteins requires further investigation. Future research using proteomics techniques could identify other potential target proteins of MdPUB30 and elucidate their functional network during drought stress.

### MYB47 and bZIP46 orchestrate differential drought responses in apple rootstocks through distinct regulatory networks

During plant responses to abiotic stress, stress-responsive gene expression depends on specific interactions between cis-acting elements in their promoter regions and TFs. These DNA–protein interactions play crucial roles in regulating stress-responsive gene expression (Liu et al. 2014a, 2014b). For example, INDUCER OF CBF EXPRESSION1 (ICE1) binds to H-box elements, ABA-responsive elements, and other cis-acting elements in its target genes, including IBS1 and E-box elements, thereby regulating the expression of genes involved in ROS scavenging (Wang et al. 2021). During biotic stress responses, ETHYLENE RESPONSE FACTOR1 (ERF1) regulates gene expression by binding to GCC boxes in its target genes, while under abiotic stress, it binds to DRE/CRT elements to upregulate specific genes in response to varying stress conditions (Cheng et al. 2013). Our analysis of the *NAC17* promoters in *M. sieversii* and M26 revealed that a single base polymorphism drives the differential drought responses between the 2 rootstocks. MYB47 positively regulates *NAC17* expression by binding to a cis-acting element in *M. sieversii*, whereas this regulation is disrupted in M26 due to the presence of a mutation (Fig. 4). This discovery highlights the role of a MYB-NAC regulatory module in plant drought resistance. MYB TFs are known to play important roles in drought responses in apple. For instance, MdMYB46 enhances tolerance to salt and osmotic stress by directly activating stress-responsive signaling (Chen et al. 2019). MdMYB44-like precisely regulates ABA-mediated salt and drought tolerance through the *MdPYL8-MdPP2CA* module (Chen et al. 2024). MdMYB88 and MdMYB124 improve drought tolerance by regulating hydraulic conductivity in roots and xylem development under prolonged drought conditions (Geng et al. 2018). However, the role of MYB47 in apple has been unclear. Our findings expand our understanding of the functional scope of the MYB-NAC regulatory network and provide insight into the molecular mechanisms underlying variations in drought resistance in apple rootstocks. Future research should explore whether MYB47 regulates additional downstream target genes involved in drought resistance and its potential roles in other abiotic stress responses. Additionally, the activities of MYB47 and NAC17 should be validated using genome-editing technologies such as CRISPR/Cas9 to facilitate their application in breeding drought-resistant apple rootstocks.

In addition to NAC and MYB TFs, bZIP family members play vital roles in plant responses to abiotic stress. In Arabidopsis, several bZIP TFs (eg, ABF1, AREB1/ABF2, ABF3, and AREB2/ABF4) participate in drought responses by regulating the ABA signaling pathway (Tu et al. 2018). In rice, the bZIP TFs TRAB1, OsbZIP23, OsbZIP46, OsbZIP72, OsbZIP12/OsABF1, and OsABI5 contribute to ABA signaling and environmental stress responses (Liu et al. 2014a, 2014b). Furthermore, ZmbZIP4 enhances abiotic stress tolerance in maize by regulating ABA biosynthesis and root development (Ma et al. 2018). GmbZIP15 increases sensitivity to salt and drought stress by negatively regulating *GmWRKY12* and *GmABF1* expression in soybean (Zhang et al. 2020). Here we showed that *PUB30* was significantly induced by drought stress in M26 but showed a minimal response in *M. sieversii* (Fig. 7a), contributing to their differences in drought resistance. Promoter sequence analysis revealed single-base mutations at −1,579 (*p1* region) and −840 (*p2* region) in *PUB30* (Figure S20a). GUS reporter assays confirmed that the mutation in the *p1* region underlies the differential expression of *PUB30* in the 2 rootstocks (Figure S20b). bZIP46 specifically binds to the *PUB30* promoter in M26, enhancing its activity (Fig. 7b and c), as validated by EMSA (Fig. 7d). *bZIP46* responded significantly to drought stress in both rootstocks (Figure S21), highlighting its role in the drought response.

**Figure 8:**
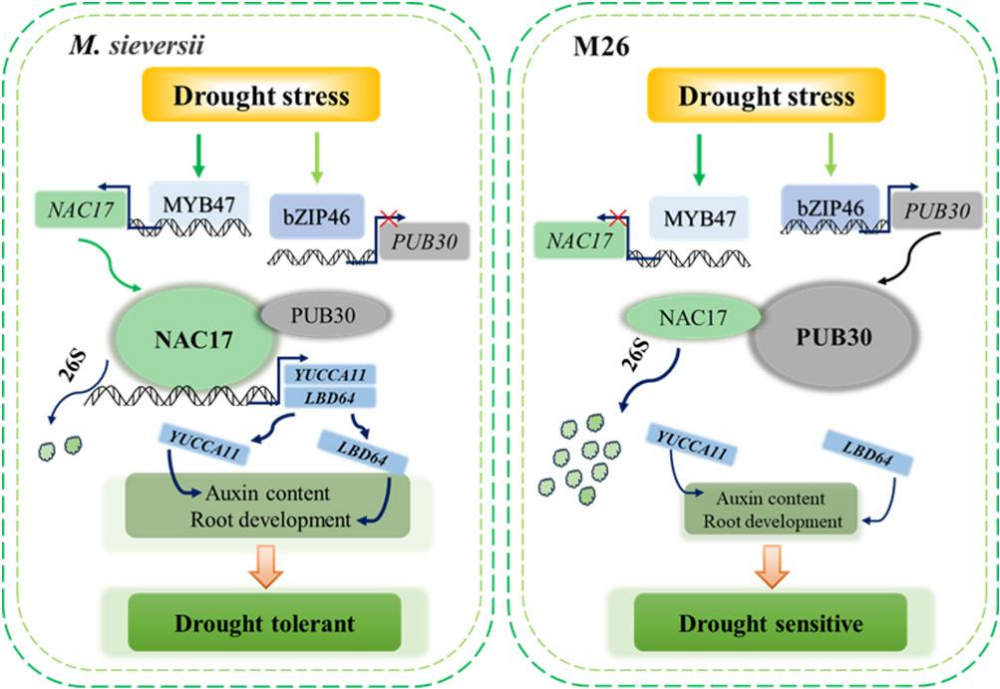
Proposed model of how the *NAC17–PUB30* module modulates the drought response by influencing adventitious root development. In the drought-resistant apple rootstock *Malus sieversii*, drought stress upregulates *MYB47* expression. In turn, MYB47 positively regulates *NAC17* expression. NAC17 promotes adventitious root development by upregulating the expression of the root development-related gene *LBD64* and the auxin biosynthesis gene *YUCCA11*, thereby enhancing drought tolerance. Additionally, drought-induced bZIP46 in *M. sieversii* does not upregulate *PUB30* expression, minimizing PUB30-mediated ubiquitination and degradation of NAC17. By contrast, in the drought-sensitive dwarfing rootstock M26, drought-induced MYB47 fails to upregulate *NAC17* expression. Meanwhile, drought-induced bZIP46 positively regulates *PUB30* expression, leading to extensive ubiquitination and degradation of NAC17. This results in reduced NAC17 protein abundance, impaired adventitious root development, and increased drought sensitivity in M26.

In conclusion, MsNAC17 enhances drought resistance in apple rootstocks by upregulating *MdLBD64* and *MdYUCCA11*, thereby promoting root development and auxin accumulation. This finding offers insights into how TFs and changes in ubiquitination coordinately regulate plant stress responses. We propose a model (Fig. 8) for the role of the *NAC17–PUB30* module in the drought-stress response in apple rootstock. MYB47 positively regulates *NAC17* expression by binding to its promoter region in *M. sieversii*, while MdPUB30 modulates NAC17 stability through ubiquitination-mediated degradation, thereby affecting the transcriptional activation of downstream target genes. Additionally, bZIP46 enhances drought sensitivity in M26 by regulating *MdPUB30* expression. These findings shed light on the role of the *NAC17–PUB30* module in the varying levels of drought resistance among apple rootstocks. A systematic evaluation should be performed on the potential value of the *NAC17–PUB30* module for the targeted improvement of drought resistance in the dwarfing apple rootstock M26, as well as on the roles of this module in other abiotic stress responses. Finally, the potential application of this module for drought-resistant apple breeding should be validated using genome-editing technologies.

## Materials and methods

### Plant materials and treatments

Wild apple (*Malus sieversii* Roem.) and the dwarfing rootstock M26 (*Malus domestica* Borkh.) were used in this study, both of which were maintained under sterile tissue culture conditions. Tissue-cultured and cutting-propagated plants were cultivated in a controlled climate chamber at 23 ± 1 °C, with a relative humidity of 30 to 40%, a 16 h/8 h light/dark cycle, and a light intensity of 200 to 300 μmol·m^−2^·s^−1^.

For PEG-induced drought treatment, cutting-propagated plants were transferred to rooting medium (MS + 0.5 mg/L IBA) after 30 d of subculture and grown for an additional 30 d. The cutting-propagated plants were then acclimated and transplanted into a hydroponic system containing 1/2 Hoagland nutrient solution (pH 5.8), followed by treatment with 20% PEG6000 (w/v) to simulate moderate drought stress. Samples were collected at 0, 2, 4, 8, 12, and 24 h, with 3 biological replicates per time point. Collected samples were flash-frozen in liquid nitrogen and stored at −80 °C for subsequent analyses.

For natural drought treatment, rooted cutting-propagated plants were transplanted into a vermiculite-soil mixture (2:3, v/v). After thorough watering, irrigation was withheld for 14 d. Shoot and root samples were collected, with well-watered plants serving as controls. Root phenotypic analysis was conducted using a root scanner (LD-GX02, Lainde, China) to measure root length, surface area, volume, and tip number. For all experiments, plant samples collected immediately before the application of drought stress are designated as “Before drought”, while those collected after the drought period are designated as “After drought”.

Seeds of tobacco (*Nicotiana benthamiana*), grown under identical conditions, were used for transient expression assays.

### Genetic transformation of apple dwarfing rootstock M26

The overexpression/antisense vector pRI101-*NAC17* and the antisense vector pRI101-*PUB30* (which carries a constitutive *35S*::GFP reporter) were constructed and introduced into the *Agrobacterium tumefaciens* strain GV3101. Bacterial cultures were grown in YEP medium containing 100 mg/L kanamycin and 50 mg/L rifampicin at 28 °C until the optical density at 600 nm (OD_600_) reached 0.6 to 0.8. Young M26 leaves were wounded, immersed in bacterial suspension, and co-cultured on MS medium with 2.0 mg/L 6-BA, 0.4 mg/L IBA, and 200 μM acetosyringone in the dark for 3 d. After washing with timentin (500 mg/L), the leaves were transferred to selection medium (MS + 2.0 mg/L 6-BA + 0.4 mg/L IBA + 500 mg/L timentin + 4 mg/L kanamycin). Resistant shoots were verified via PCR and propagated for functional analysis.

### RNA extraction, cDNA synthesis, and RT-qPCR analysis

Total RNA was extracted using the TRNzol Universal RNA Extraction Kit (Tiangen Biotech, China). RNA purity and integrity were assessed using a NanoDrop 2000 spectrophotometer (Thermo Fisher, USA) and 1% agarose gel electrophoresis. cDNA was synthesized using the HiScript II 1st Strand cDNA Synthesis Kit (Vazyme, China). RT-qPCR was performed on a Rotor-Gene Q system (Qiagen, Germany) using 2×HQ SYBR qPCR Mix (Zhuangmeng, China). The apple *Actin* gene (GenBank: XM_008393049.4) and *Histone H3* (GenBank: MD08G1187800) served as the internal controls, and relative expression levels were calculated using the 2^−ΔΔCt^ method. All the primers used are listed in Table S2.

### Subcellular localization analysis

The *MsNAC17* coding sequence (excluding the stop codon) was cloned into the pCAMBIA1302-GFP vector and introduced into *Agrobacterium* GV3101. Bacterial cultures (OD_600_ = 0.9 to 1.0) were then infiltrated into *N. benthamiana* leaves alongside the nuclear marker mCherry GFP (488/507 nm), and mCherry (587/610 nm) signals were visualized using a confocal laser scanning microscope (LSM 880, Zeiss, Germany).

### Drought survival assessment

Survival of plantlets under drought stress was assessed at 0, 7, and 14 d of treatment based on morphological criteria. Plantlets were scored as dead if they exhibited complete loss of turgor, total leaf desiccation, and browning or necrosis of the basal meristematic tissue. Those retaining any visible green tissue, particularly in the apical meristem or leaf sheaths, were considered alive. The survival rate at each time point was calculated as a percentage of the initial number of plantlets per treatment.

### Soil water content determination

Soil samples were collected from the root zone of each plant at 0, 7, and 14 d after the initiation of the natural drought treatment. The gravimetric water content was determined using the standard oven-drying method. Fresh soil was immediately placed into pre-weighed aluminum cans, sealed, and weighed. The samples were then oven-dried at 105 °C for 48 hours to constant weight. After cooling in a desiccator, the dry weight was recorded. Soil water content was calculated as follows: SWC (%) = [(fresh weight – dry weight)/(dry weight – can weight)] × 100.

### Measurement of physiological indicators

All analytical methods for the measured parameters in this study followed the standard protocols established by Liu et al. (Liu et al. 2021). Physiological characterization was conducted on intact soil-grown apple plants (including both aerial and root tissues) using the following analytical approaches with commercial kits (Solarbio, China): malondialdehyde (MDA) content was quantified by the thiobarbituric acid (TBA) method; proline concentration was determined via the sulfosalicylic acid-ninhydrin assay; superoxide anion was measured by hydroxylamine oxidation; hydrogen peroxide (H_2_O_2_) was measured by the titanium sulfate method; and the activities of catalase (CAT), superoxide dismutase (SOD), peroxidase (POD), and ascorbate peroxidase (APX) were assessed using antioxidant enzyme activity assays. Six biological replicates (independent plant individuals) were performed, with 3 technical replicates per sample to ensure methodological rigor.

### Indole-3-acetic acid content measurement

Fresh root tissue (0.5 g) was homogenized in 2 mL of ice-cold extraction buffer (80% methanol with 1% glacial acetic acid) and centrifuged at 12,000 ×g for 10 min at 4 °C. The supernatant was purified using a C18 solid-phase extraction column (Waters, USA). Indole-3-acetic acid (IAA) was eluted with 2 mL of 80% methanol containing 1% glacial acetic acid, dried under nitrogen, and reconstituted in 100 μL of PBS (pH 7.4). IAA content was quantified using an ELISA kit (Agrisera, Sweden) following the instructions from the manufacturer.

### Yeast hybrid assays

Total RNA was extracted from *Malus domestica* subjected to drought stress and reverse-transcribed into cDNA. The cDNA fragments were then ligated into yeast two-hybrid vectors pGADT7 using homologous recombination or Gateway® cloning technology. The recombinant plasmids were transformed into competent yeast cells via electroporation (performed by OE Biotech Co., Ltd.). Positive clones were selected on SD/−Leu/−Trp dropout medium, and the library quality was assessed through colony PCR and sequencing to confirm insert size and coverage. For yeast one-hybrid screening, the *NAC17* and *PUB30* promoters were cloned into the pAbAi vector and transformed into Y1HGold yeast. Positive clones were selected on SD/−Leu/AbA medium and confirmed through colony PCR and sequencing. For yeast two-hybrid screening, the *NAC17* and *PUB30* coding regions were cloned into pGBKT7 (BD) and pGADT7 (AD) vectors, respectively. Co-transformed Y2HGold cells were plated on SD/−Trp/−Leu/X-α-Gal/AbA medium. Positive clones were verified using colony PCR and sequencing.

### Dual-luciferase reporter assay

*NAC17*, *MYB47*, and *bZIP46* coding regions of apple were cloned into the pGREEN-62-SK effector vector, and the −2.0 kb promoter regions of *proNAC17*, *proPUB30*, *proMdLBD64* or *proMdYUCCA11* were cloned into the pGREEN-0800-LUC reporter vector. *Agrobacterium* GV3101 cultures (OD_600_ = 0.6) were then infiltrated into *N. benthamiana* leaves. LUC fluorescence signals were captured using an IVIS Lumina III imaging system (PerkinElmer, USA).

### Promoter activity analysis

The *proNAC17* and *proPUB30* promoters (−2.0 kb) were respectively cloned into the pCAMBIA1301 vector (replacing the 35S promoter) to generate GUS fusion vectors. *Agrobacterium* GV3101 (OD_600_ = 0.8) was then infiltrated into *Nicotiana benthamiana* leaves, and GUS activity was measured following PEG-induced drought stress.

### Electrophoretic mobility shift assay

*NAC17*, *MYB47*, and *bZIP46* coding regions were cloned into the pGEX-4T-1 vector (GST tag) and expressed in *E. coli* BL21 (DE3). Proteins were purified using GST affinity chromatography. Biotin-labeled probes were synthesized based on predicted cis-acting elements. DNA–protein complexes were separated on a 6% non-denaturing polyacrylamide gel and detected using a chemiluminescent electrophoretic mobility shift assay (EMSA) kit (Thermo Fisher, USA).

### Pulldown assay

The coding regions of *MdPUB30* and *MsNAC17* were cloned into the pET-30a (His tag) and pGEX-4T-1 (GST tag) vectors, respectively. Proteins were expressed in *E. coli* BL21 (DE3) and purified using Ni-NTA and GST affinity chromatography. His-MdPUB30 and GST-MsNAC17 were incubated in binding buffer (20 mM Tris-HCl, 100 mM NaCl, 1 mM EDTA, 0.5% NP-40, pH 7.5) at 4 °C for 4 h. After incubation, bound proteins were eluted and analyzed using Western blot with an anti-GST antibody (1:5000, Abcam).

### Ubiquitination assays in vitro and in vivo

For the in vitro ubiquitination assay, the reaction mixture (50 μL) consisted of E1 (50 nM), UbcH5a (100 nM), His-PUB30 (200 nM), GST-NAC17 (2 μg), ubiquitin (5 μg), ATP (2 mM), and reaction buffer (50 mM Tris-HCl, 5 mM MgCl_2_, 2 mM DTT, pH 7.5). After incubation at 30 °C for 2 h, the reaction products were resolved by SDS-PAGE and detected via Western blot using anti-ubiquitin and anti-GST antibodies. For the in vivo assay, NAC17-GFP and PUB30-His were transiently expressed in *Agrobacterium* GV3101 and co-infiltrated into *N. benthamiana* leaves. Total proteins were extracted using RIPA buffer and subjected to immunoblot analysis with anti-GFP and anti-ubiquitin antibodies to assess ubiquitination levels.

### Statistical analysis

The data were analyzed using one-way ANOVA followed by Tukey's honestly significant difference (HSD) post hoc test for multiple comparisons among 3 or more groups. For experiments involving 2 factors, two-way ANOVA with Sidak and Tukey's multiple comparisons test was applied. Comparisons between 2 groups at a single experimental point were performed using Student's *t*-test. All analyses were conducted with GraphPad Prism 9.3.1 (GraphPad Software, LLC, USA). Results are expressed as means ± SEM. Significant differences are indicated by asterisks (*P < 0.05, **P < 0.01) or distinct lowercase letters (for multiple comparisons following ANOVA). All experiments were independently repeated at least 3 times.

### Accession numbers

Sequence data from this article can be found in the GenBank/EMBL data libraries under accession numbers XM_050249457.2 (*NAC17*), XM_070815379.1 (*PUB30*), XM_008349892.4 (*MdLBD64*), XM_050251850.2 (*MdYUCCA11*), XM_008377801.4 (*MYB47*), XM_070812349.1 (*bZIP46*).

## Supplementary Material

kiag488_Supplementary_Data

## Data Availability

The data that support the findings of this study are available from the corresponding author upon reasonable request.
