# Supplementary material for: The NAC17–PUB30 module enhances drought stress tolerance by regulating adventitious root development in apple
Source: Plant Physiol. 2026 Jul 10;201(3):kiag488. doi: 10.1093/plphys/kiag488 (PMC13418357; doi:10.1093/plphys/kiag488)
Supplement: kiag488_Supplementary_Data [file kiag488_supplementary_data.zip › Supplementary Data (5).pdf]

Supplementary Figures

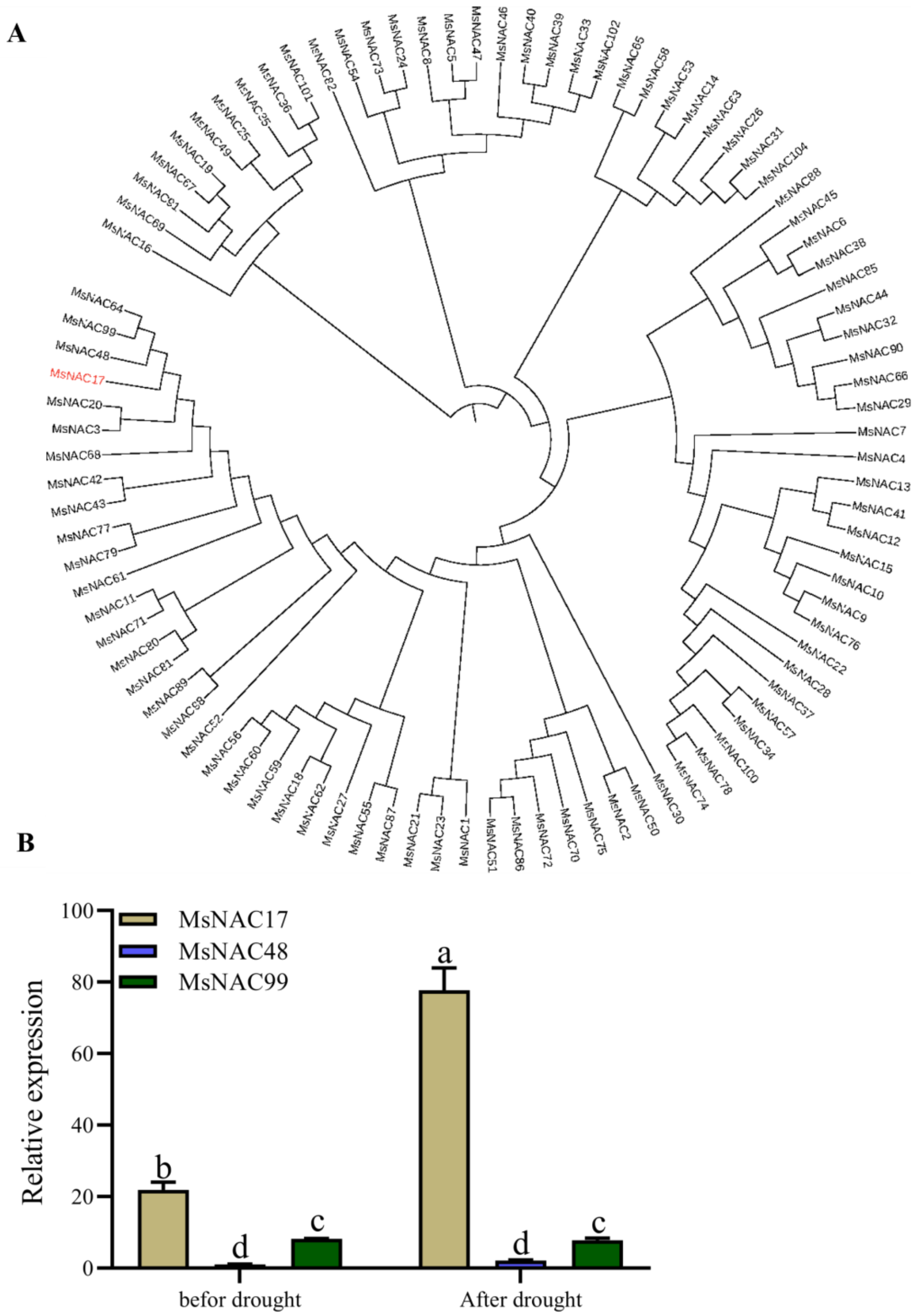

Supplementary Figure S1. Phylogenetic tree and drought expression analysis of the MsNAC transcription factor family. **A,** Phylogenetic tree of NAC proteins in *Malus sieversii*. Red font indicates MsNAC17. The phylogenetic tree was constructed by first performing multiple sequence alignment of the MsNACs protein sequences using ClustalW, followed by the neighbor-joining method in MEGA 11. **B,** Relative expression analysis of *NAC17* homologs

under drought stress. Error bars represent standard deviation (SD) of three biological replicates ($n = 3$). Student's t-test was used to evaluate statistical significance, with different lowercase letters indicating significant differences at $P < 0.05$.

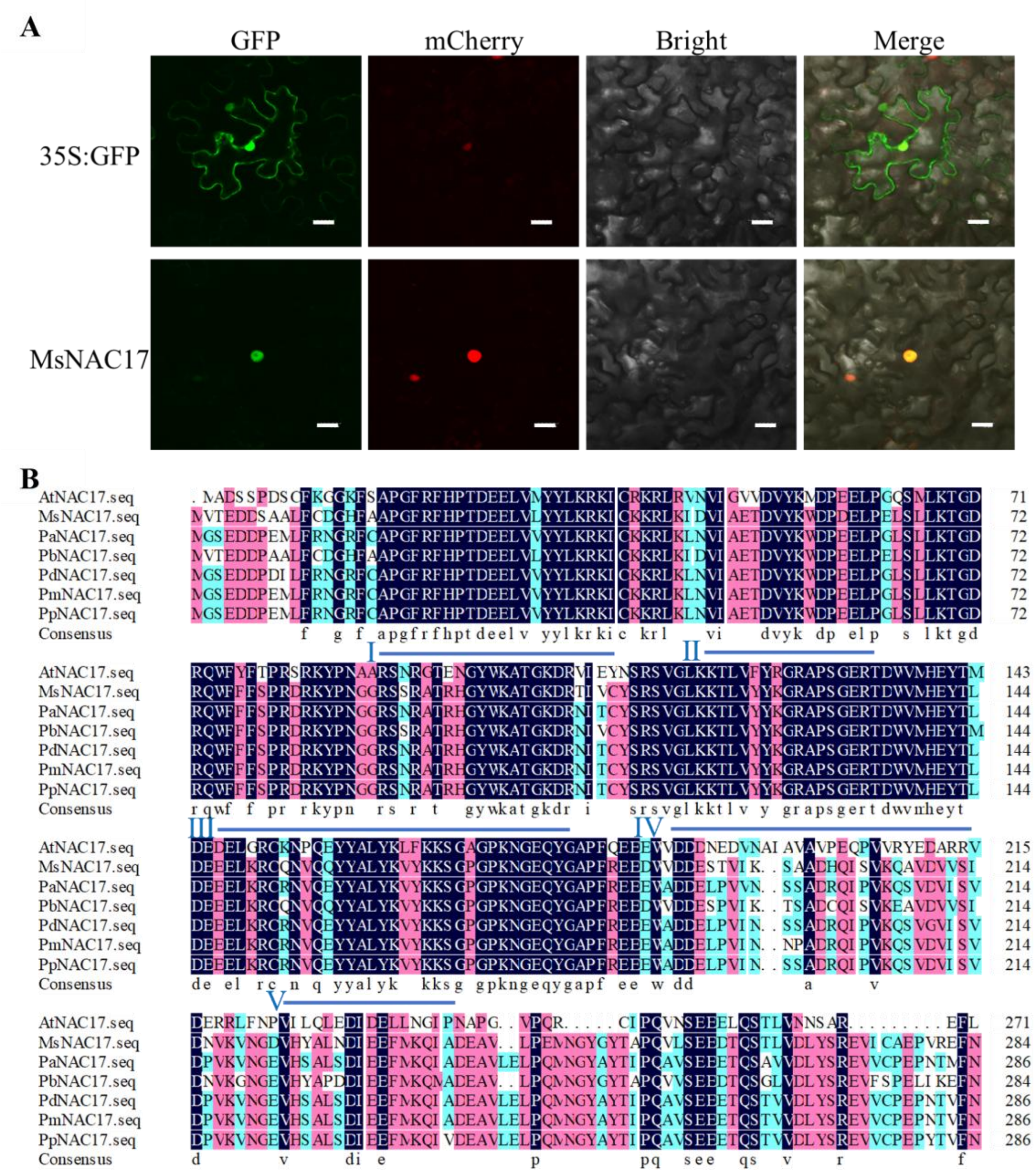

Supplementary Figure S2. Subcellular localization and domain analysis of MsNAC17. **A**, Subcellular localization of the NAC17-GFP fusion protein in *Nicotiana benthamiana* leaf epidermal cells. The MsNAC17-GFP fluorescence signal is restricted to the nucleus, while GFP control (empty vector) shows fluorescence distributed throughout the cell. Scale bar, 20 μm. **B**, Amino acid sequence alignment of NAC proteins from *Malus sieversii* (MsNAC17,

XP_028965254.1), *Arabidopsis thaliana* (AtNAC17, NP_564440.1), *Prunus avium* (PaNAC17, XP_021820479.1), *Pyrus bretschneideri* (PbNAC17, XP_009339761.2), *Prunus dulcis* (PdNAC17, XP_034228200.1), *Prunus mume* (PmNAC17, XP_008237396.1), and *Prunus persica* (PpNAC17, XP_007201176.1). Five conserved subdomains (I–V) are indicated with blue lines. Sequence alignment was performed using DNAMAN 9.0.

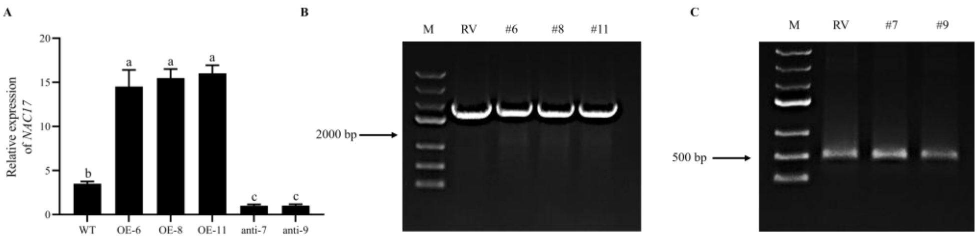

Supplementary Figure S3. Identification of *MsNAC17* transgenic materials. **A**, Relative expression levels of *NAC17* in *MsNAC17*-overexpression and -silencing lines. Data are presented as the mean ± SD of three biological replicates. Different letters indicating significant differences ($p<0.05$, one-way ANOVA with Tukey's HSD test). **B**, DNA-level identification of *MsNAC17* overexpression lines by agarose gel electrophoresis. **C**, DNA-level identification of *MsNAC17* silencing lines by agarose gel electrophoresis. Abbreviations, M: Marker, RV: Recombinant vector, #6: *MsNAC17*-OE-6, #8: *MsNAC17*-OE-8, #11: *MsNAC17*-OE-11, #7: *MsNAC17*-anti-7, #9: *MsNAC17*-anti-9.

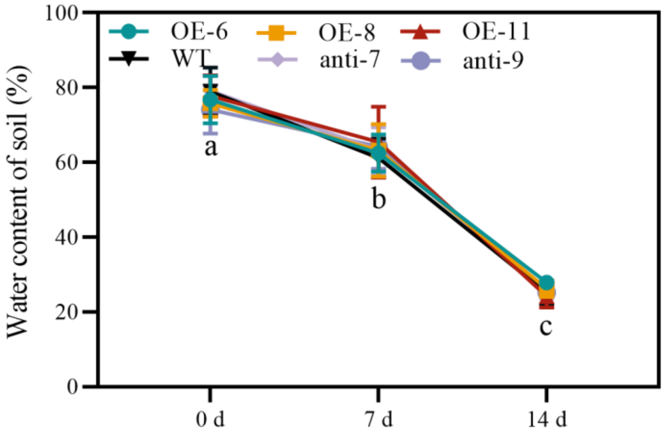

Supplementary Figure S4. Dynamics of soil water content in pots containing plants of different genotypes during the 14 d drought stress period. OE-6, OE-8 and OE-11 denote three independent *MsNAC17*-overexpressing lines in the M26 background, while anti-7 and anti-9

represent two *MsNAC17*-silenced lines. Values are means ± SD (n = 3 biological replicates). Statistical significance was determined by one-way ANOVA followed by Tukey's post hoc test ($p < 0.05$). Different lowercase letters indicate significant differences among genotypes at the same time point.

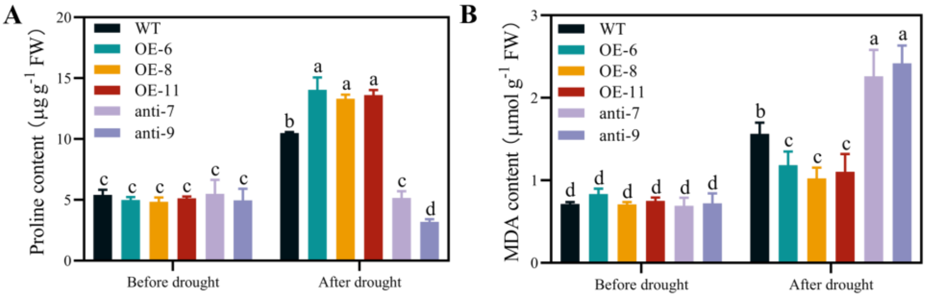

Supplementary Figure S5. Physiological indices in *MsNAC17* transgenic lines. **A**, Proline content in *MsNAC17* transgenic and wild-type plants under drought conditions. **B**, MDA content in different genotypes during drought stress. Error bars represent the mean ± SE (n = 10); Different letters indicating significant differences (p<0.05, one-way ANOVA with Tukey's HSD test). OE-6, OE-8 and OE-11 denote three independent *MsNAC17*-overexpressing lines in the M26 background, while anti-7 and anti-9 represent two *MsNAC17*-silenced lines. Plants were subjected to 14 days of natural drought treatment under field conditions. Abbreviations: WT, wild type.

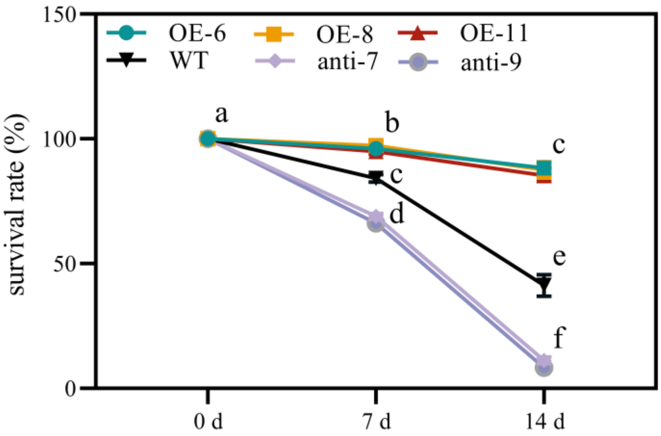

Supplementary Figure S6. Statistical analysis of survival rates of various transgenic strains under drought stress. Error bars represent the mean ± SE (n = 10); Different letters indicating significant differences (p<0.05, one-way ANOVA with Tukey's HSD test). OE-6, OE-8 and

OE-11 denote three independent *MsNAC17* overexpressing lines in the M26 background, while anti-7 and anti-9 represent two *MsNAC17* silenced lines. Plants were subjected to 14 days of natural drought treatment under field conditions. The sampling times were 0 days, 7 days, and 14 days of natural drought treatment, respectively. Abbreviations: WT, wild type.

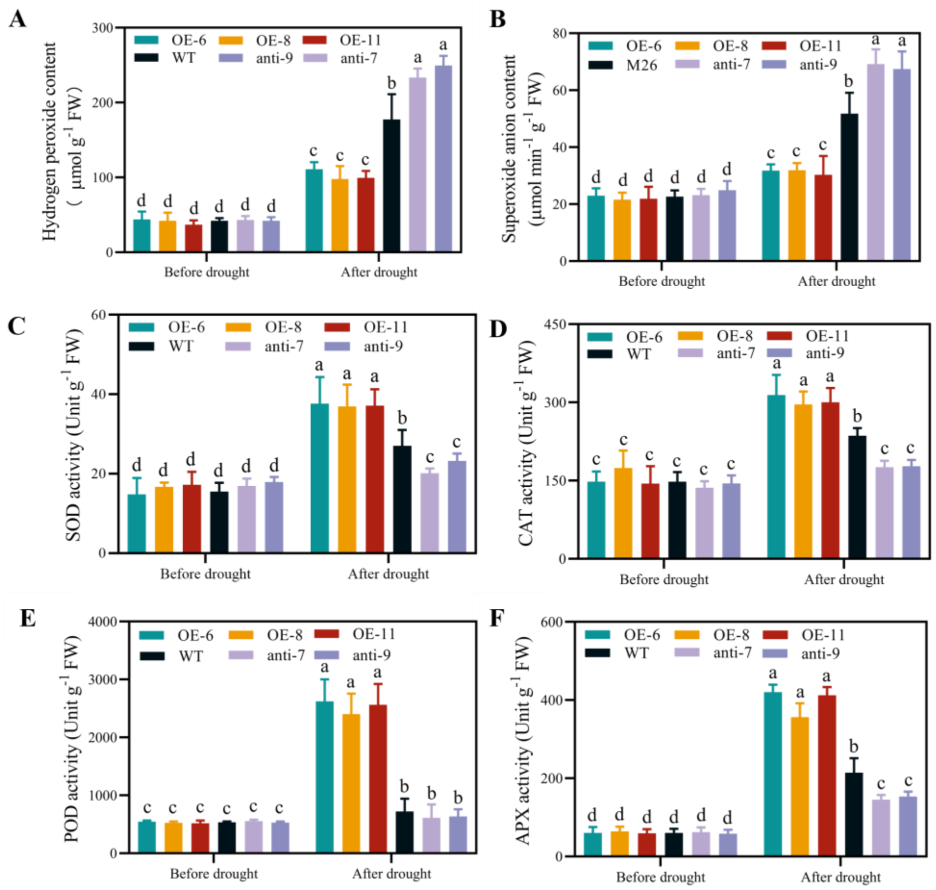

Supplementary Figure S7. Reactive oxygen species (ROS) content and antioxidant enzyme activity in *MsNAC17*-overexpressing and *MsNAC17*-silenced lines under drought stress. **A**, Hydrogen peroxide content. **B**, Superoxide anion content. **C**, Superoxide dismutase (SOD) activity assay. **D**, Catalase (CAT) activity assay. **E**, Peroxidase (POD) activity assay. **F**, Ascorbate peroxidase (APX) activity assay. Error bars represent the mean ± SE (n = 10); Different letters indicating significant differences (p<0.05, one-way ANOVA with Tukey's HSD test). OE-6, OE-8 and OE-11 denote three independent *MsNAC17*-overexpressing lines in the M26 background, while anti-7 and anti-9 represent two *MsNAC17*-silenced lines. Plants were subjected to 14 days of natural drought treatment under field conditions. Abbreviations: WT, wild type.

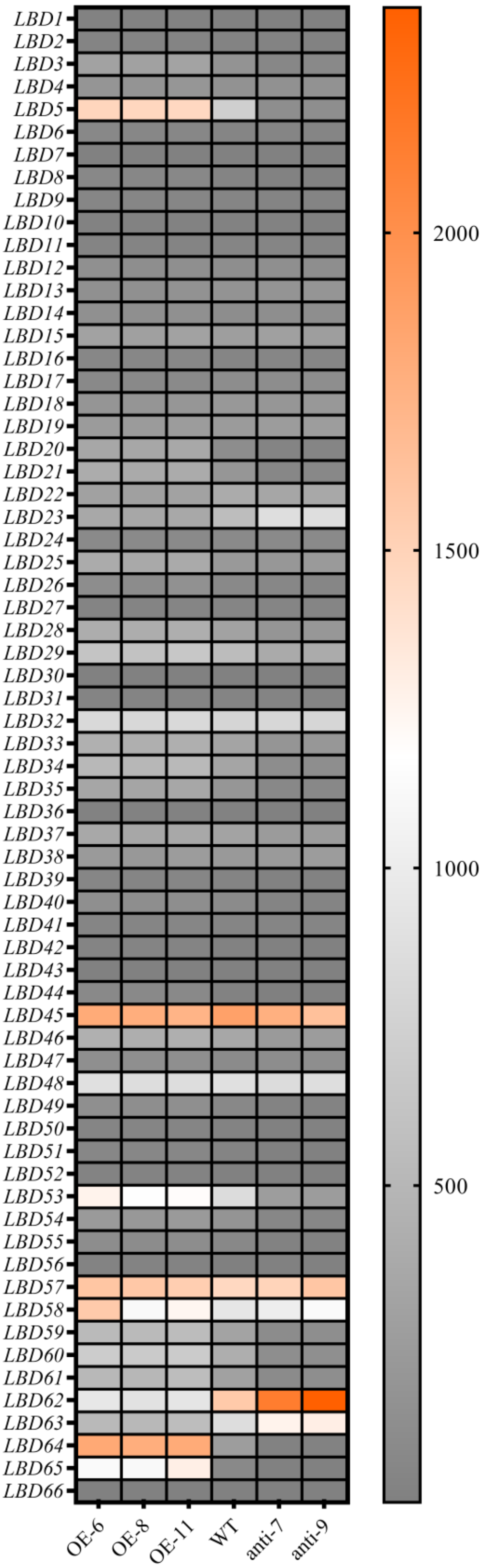

Supplementary Figure S8. Heatmap showing *MdLBD* family gene expression levels in *MsNAC17*-overexpressing and *MsNAC17*-silenced lines. The color bar represents relative

expression levels from 0 (gray, low expression) to 2500 (orange, high expression). OE-6, OE-8 and OE-11 denote three independent *MsNAC17*-overexpressing lines in the M26 background, while anti-7 and anti-9 represent two *MsNAC17*-silenced lines. Plants were subjected to 14 d of natural drought treatment under field conditions. Abbreviations: WT, wild type.

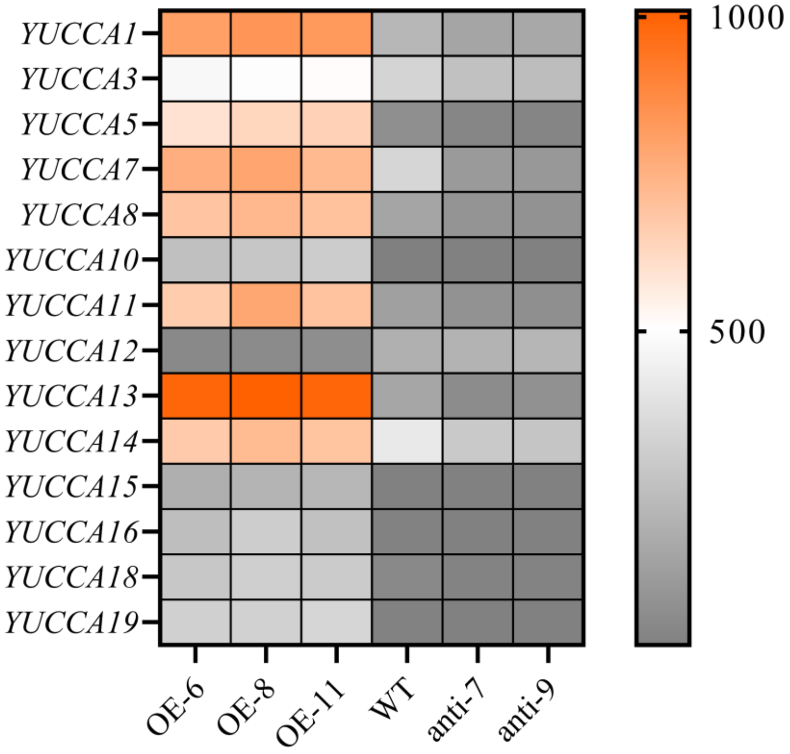

Supplementary Figure S9. *MdYUCCA* family gene expression levels in *MsNAC17*-overexpressing and *MsNAC17*-silenced lines. The color bar represents relative expression levels from 0 (gray, low expression) to 1000 (orange, high expression). OE-6, OE-8 and OE-11 denote three independent *MsNAC17*-overexpressing lines in the M26 background, while anti-7 and anti-9 represent two *MsNAC17*-silenced lines. Plants were subjected to 14 d of natural drought treatment under field conditions. Abbreviations: WT, wild type

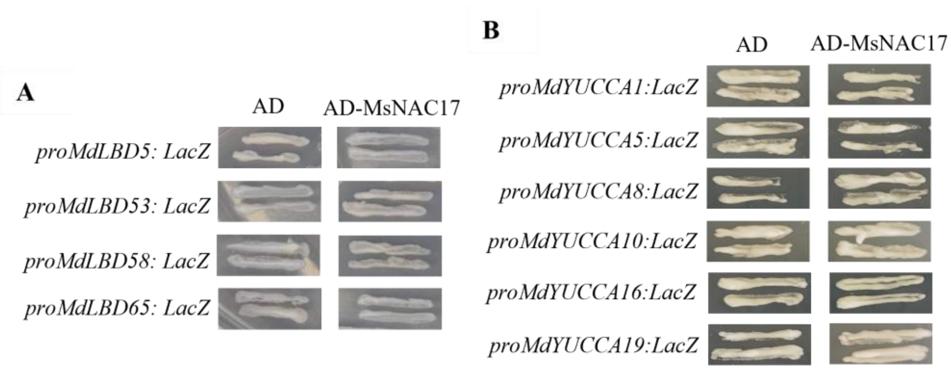

Supplementary Figure S10. Yeast one-hybrid assay showing that MsNAC17 does not bind to the promoter regions of *MdLBDs* or *MdYUCCAs* (−2.0 kb). **A,** MsNAC17 does not bind to the promoter regions of other *MdLBDs* tested. **B,** MsNAC17 does not bind to the promoter regions of other *MdYUCCAs* tested. All *LBDs* and *MdYUCCAs* were upregulated in *MsNAC17*-overexpressing lines and downregulated in *MsNAC17*-silenced lines.

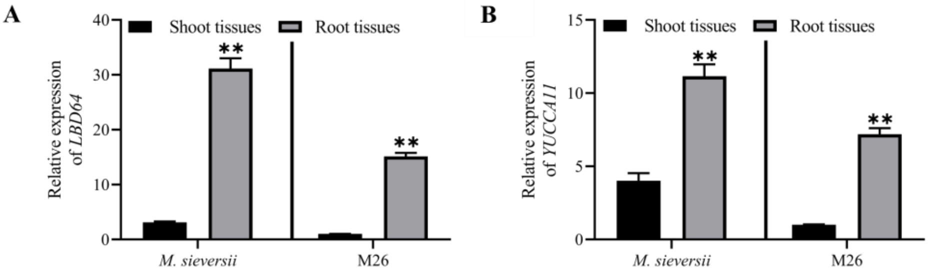

Supplementary Figure S11. Tissue-specific expression patterns of *LBD64* and *YUCCA11* in *M. sieversii* and M26 rootstocks. **A and B**, Tissue-specific expression patterns of *LBD64* and *YUCCA11* in *M. sieversii* and M26 rootstocks. Root tissues and Shoot tissues were sampled for RT-qPCR. Data are presented as mean ± SD (n = 3 biological replicates). $^{**}P < 0.01$ (two-tailed unpaired Student's t-test).

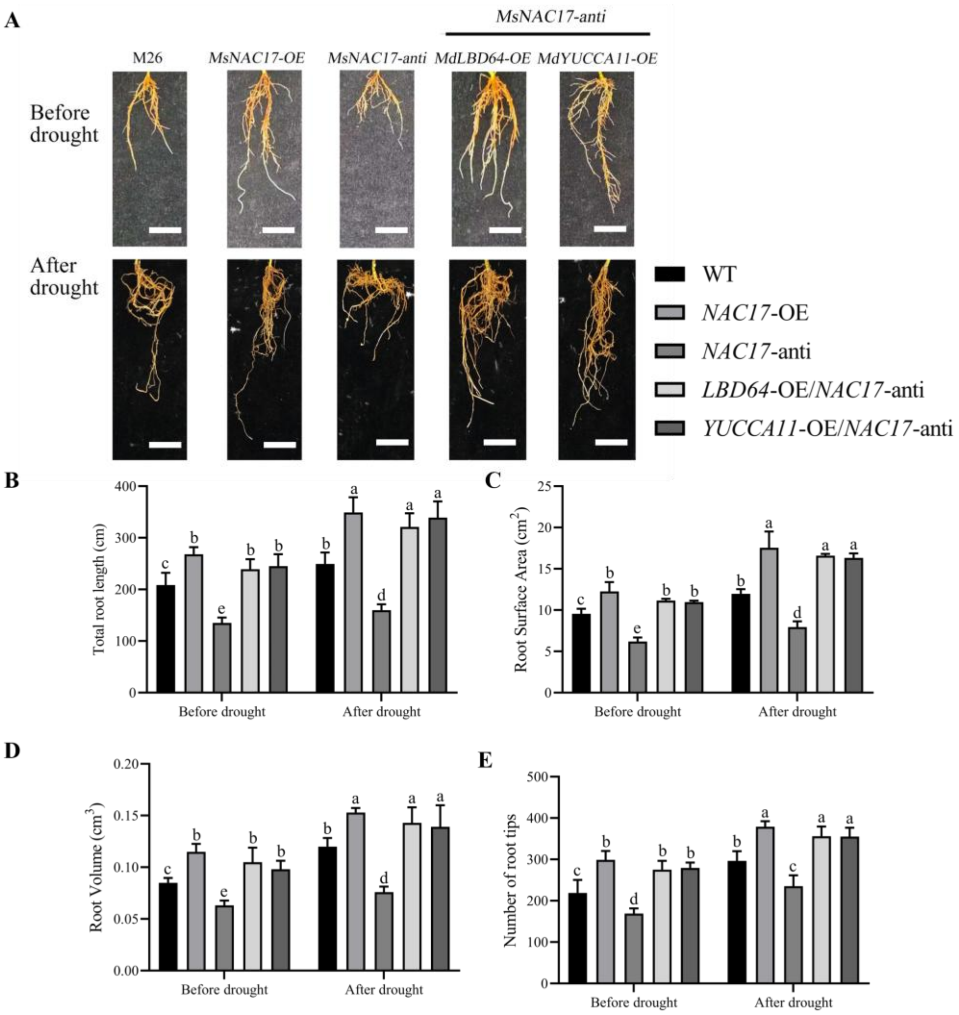

Supplementary Figure S12. Phenotypic comparison of root architecture among different genotypes under drought stress. **A,** Representative images of adventitious roots from wild-type (WT), *NAC17*-OE, *NAC17*-anti, *LBD64*-OE/*NAC17*-anti, and *YUCCA11*-OE/*NAC17*-anti plants after 14 d of natural drought treatment. Scale bar = 5 cm. **B–E,** Quantitative analysis of (B) total root length, (C) root surface area, (D) root volume, and (E) number of root tip in the indicated genotypes under drought stress conditions. All plants were subjected to 14 d of natural drought stress in soil-grown conditions. Data are presented as means ± SEM (*n* = 10 biological replicates per genotype). Different lowercase letters above the bars indicate significant differences ($p < 0.05$, one-way ANOVA with Tukey's HSD test). OE, overexpression; anti, antisense silencing.

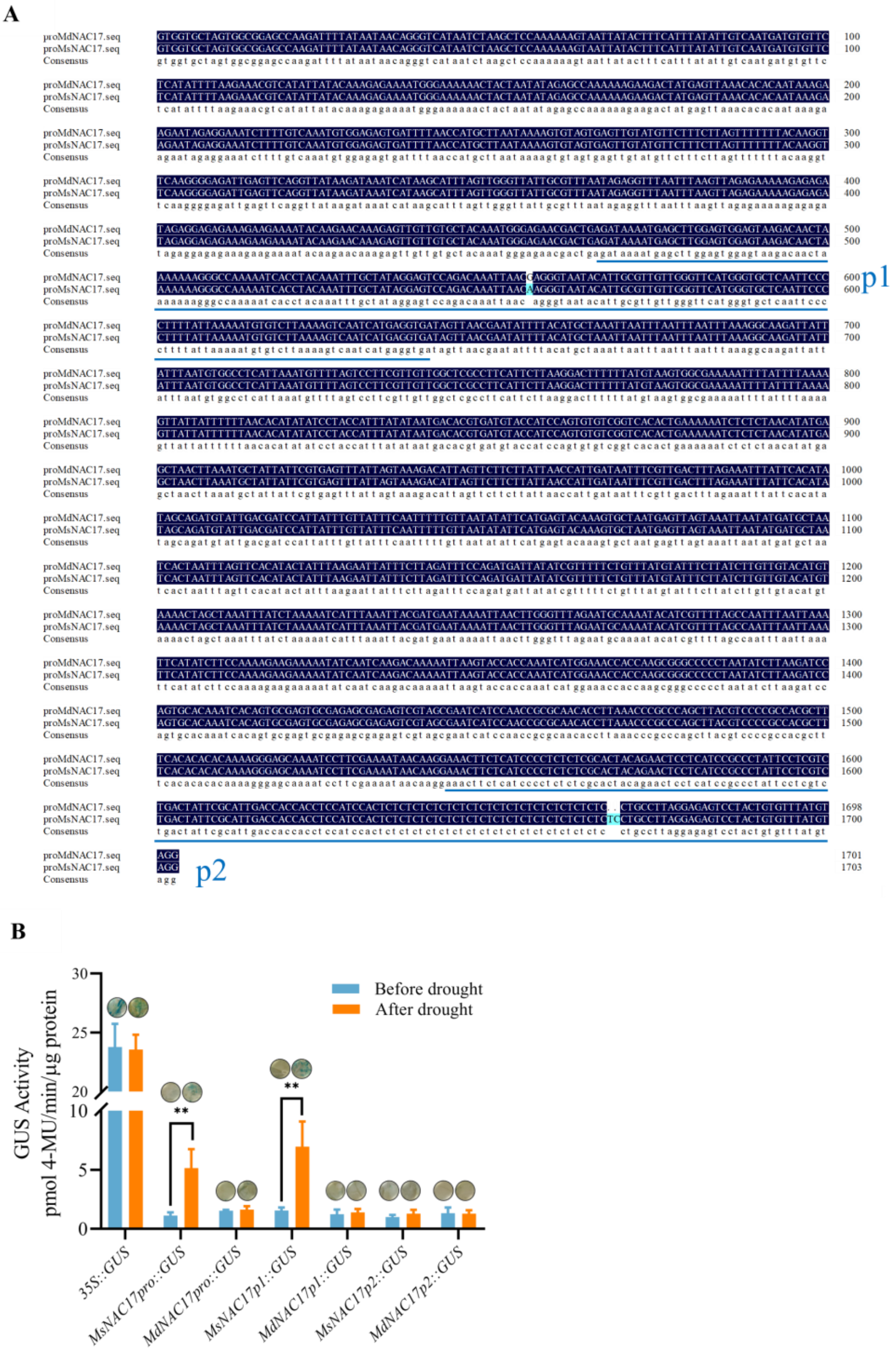

Supplementary Figure S13. Sequence alignment and GUS reporter assay of *NAC17* promoters from *M. sieversii* and M26 under drought stress. **A,** Sequence alignment of the *NAC17* promoter regions in *Malus sieversii* and M26 using DNAMAN 9.0. Blue lines indicate the p1 and p2 regions. **B,** GUS activity assay of the *NAC17* promoter in *Malus sieversii* and M26. Data are presented as the mean ± SE of three biological replicates. Significance was analyzed using Student's *t*-test, $^{**}P < 0.01$.

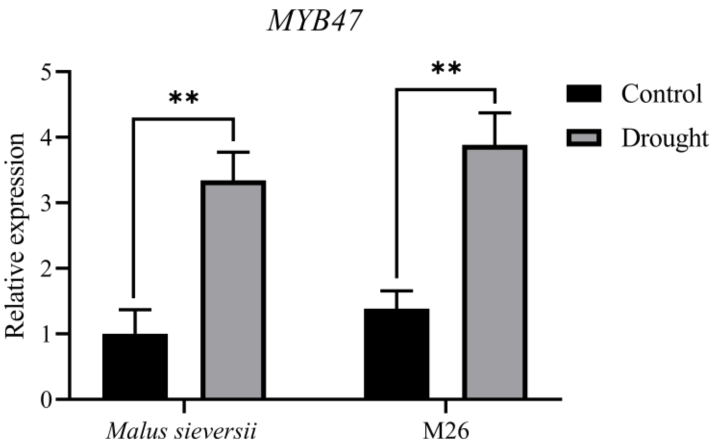

Supplementary Figure S14. Relative expression levels of *MYB47* in *Malus sieversii* and M26 under drought stress. Data are presented as the mean ± SD of three biological replicates. Student's *t*-test was used to evaluate statistical significance. $^{**}P < 0.01$.

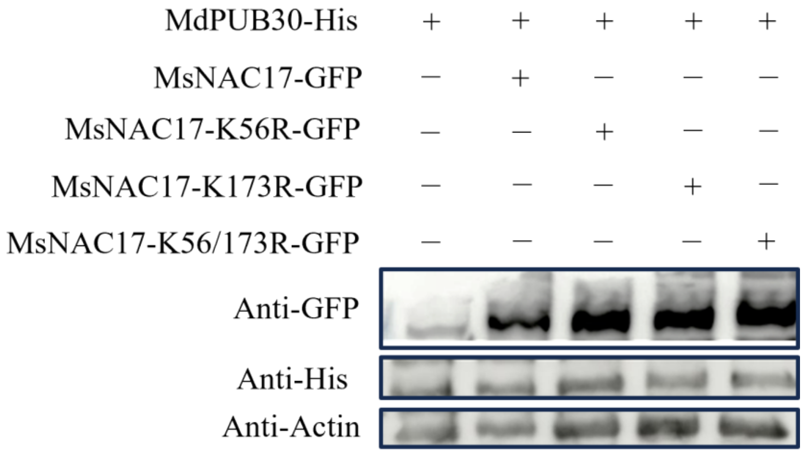

Supplementary Figure S15. Identification of ubiquitination sites in MsNAC17 for degradation. In vivo degradation assay of MsNAC17 ubiquitination-site mutants. MsNAC17 or its lysine-to-arginine mutants (K56R, K173R, and the double mutant K56/173R) were co-expressed with the E3 ligase PUB30 in *Nicotiana benthamiana* leaves.

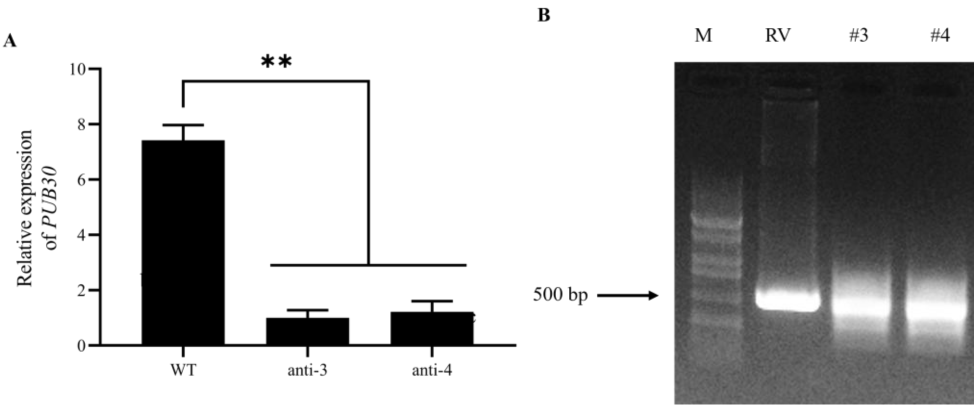

Supplementary Figure S16. Identification of *MdPUB30* transgenic materials. **A**, Relative expression levels of *PUB30*-silenced transgenic lines. Data are presented as the mean ± SD of three biological replicates. ** indicates significant difference between WT and each independent silenced line (anti-3 and anti-4) (**$p < 0.01$ for both comparisons, Dunnett's test). **B**, DNA-level identification of *MdPUB30* silencing lines by agarose gel electrophoresis. Abbreviations, M: Marker, RV: Recombinant vector, #3: *MdPUB30*-anti-3, #4: *MdPUB30*-anti-4.

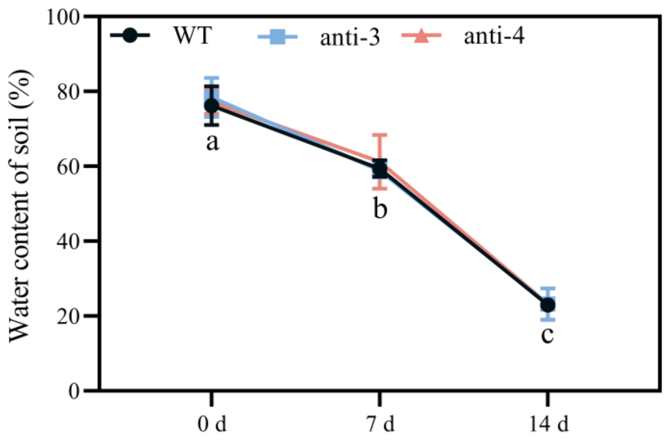

Supplementary Figure S17. Dynamics of soil water content in pots containing plants of different genotypes during the 14 d drought stress period. Anti-3 and anti-4 represent two *PUB30*-silenced lines. Values are means ± SD (n = 3 biological replicates). Statistical significance was determined by one-way ANOVA followed by Tukey's post hoc test ($p < 0.05$). Different lowercase letters indicate significant differences among genotypes at the same time point.

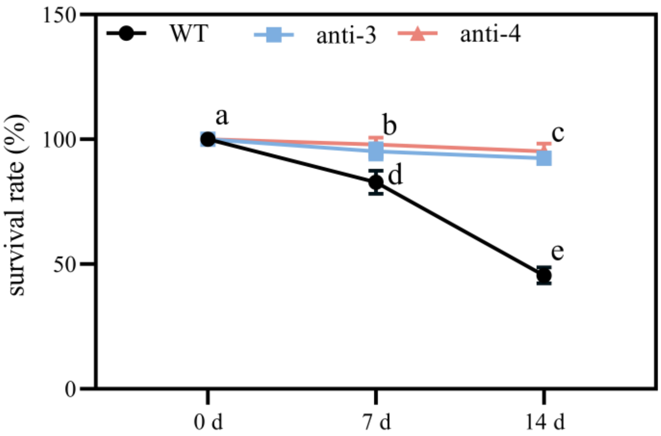

Supplementary Figure S18. Statistical analysis of survival rates of various transgenic strains under drought stress. Plants were acclimated in soil for 6 weeks after transplanting before the start of drought treatment. Error bars represent the mean ± SE (n = 10); Different letters indicating significant differences (p<0.05, one-way ANOVA with Tukey's HSD test). anti-3 and anti-4 represent two *MdPUB30*-silenced lines. Plants were subjected to 14 days of natural drought treatment under field conditions. The sampling times were 0 days, 7 days, and 14 days of natural drought treatment, respectively. Abbreviations: WT, wild type.

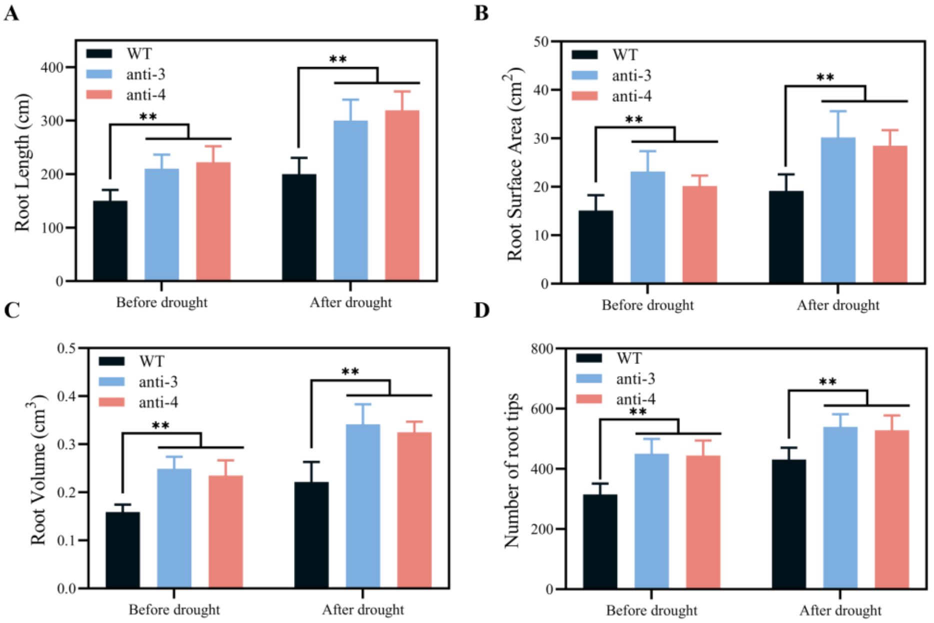

Supplementary Figure S19. Statistical analysis of total adventitious root length, root surface area, total root volume, and root tip number in various genotypes subjected to drought stress.

A to D, Statistical analysis of total adventitious root length, root surface area, total root volume, and root tip number in various genotypes subjected to drought stress. Error bars represent the mean ± SE (n = 10). Asterisks indicate significant differences between WT and silenced plants within the same treatment condition (**$p < 0.01$, Dunnett's test). Anti-3 and anti-4 represent two *PUB30*-silenced lines. Plants were subjected to 14 d of natural drought treatment under field conditions. Abbreviations: WT, wild type. **$p < 0.05$.

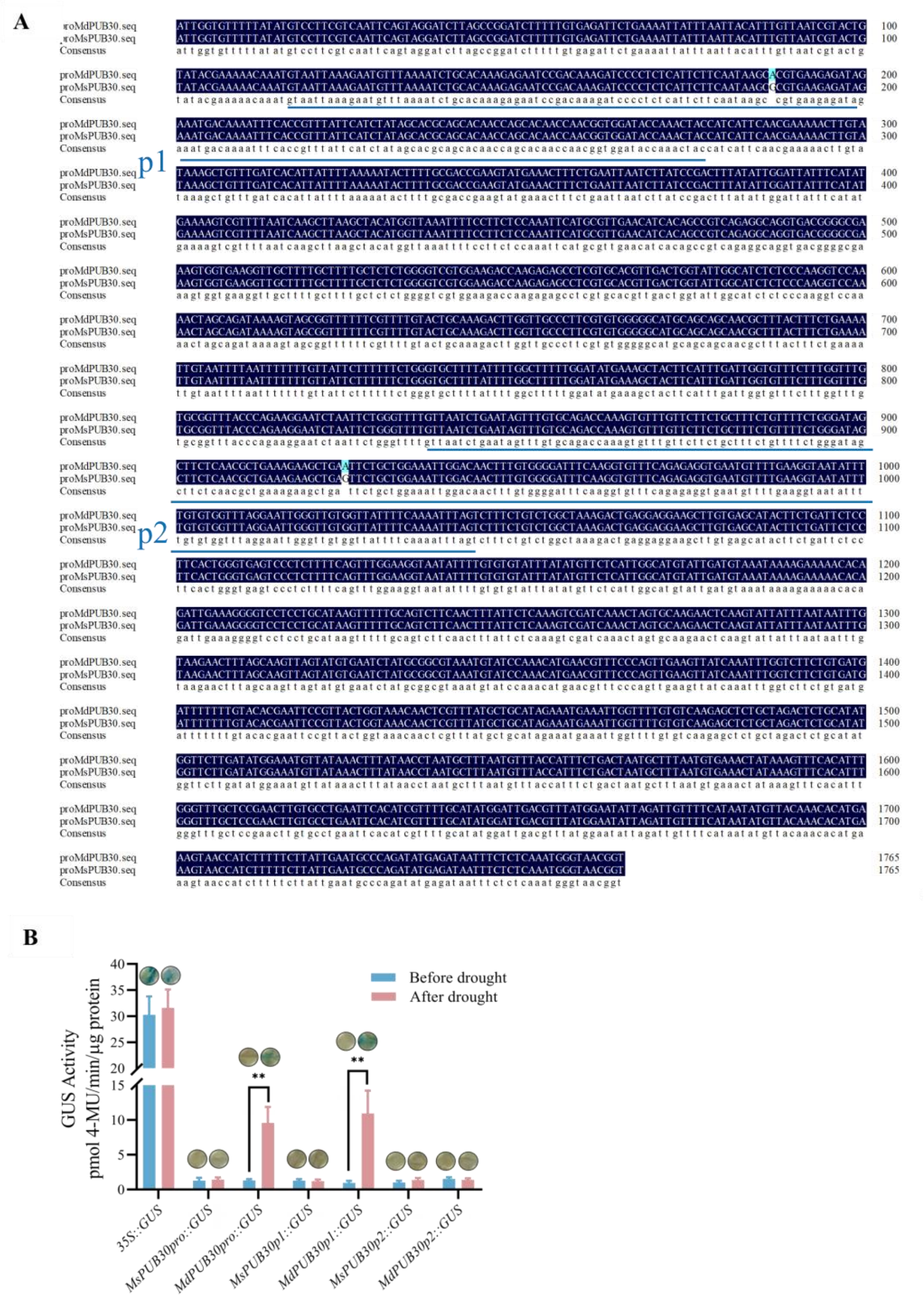

Supplementary Figure S20. Sequence alignment and GUS reporter assay of *PUB30* promoters from *M. sieversii* and M26 under drought stress. **A,** Sequence alignment of the *PUB30* promoter regions in *Malus sieversii* and M26 using DNAMAN 9.0. **B,** GUS activity assay of the *PUB30* promoter in *Malus sieversii* and M26. Data are presented as the mean ± SE of three biological replicates. Significance was analyzed using Student's *t*-test, $**P < 0.01$.

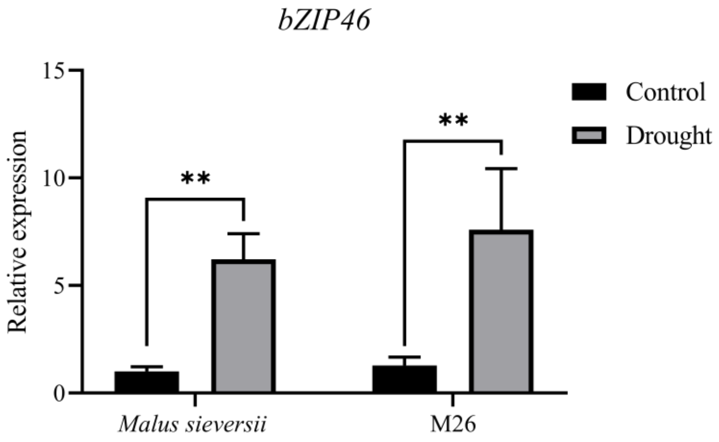

Supplementary Figure S21. Relative expression levels of *bZIP46* in *Malus sieversii* and M26 under drought stress. Data are presented as the mean ± SD of three biological replicates. Asterisks indicate significant differences between control and drought treatment condition within the same stock ($**p < 0.01$, Dunnett's test).

Supplementary Table S1 primers

Prediction of potential ubiquitination sites on the MsNAC17 protein

ID	Position	Residue	PTMscores	Cutoff=0.55
MsNAC17	36	K	Ubiquitination:0.268	None
MsNAC17	38	K	Ubiquitination:0.184	None
MsNAC17	41	K	Ubiquitination:0.151	None
MsNAC17	42	K	Ubiquitination:0.199	None
MsNAC17	45	K	Ubiquitination:0.288	None
MsNAC17	56	K	Ubiquitination:0.566	Ubiquitination:0.566
MsNAC17	69	K	Ubiquitination:0.486	None
MsNAC17	84	K	Ubiquitination:0.534	None
MsNAC17	101	K	Ubiquitination:0.325	None
MsNAC17	105	K	Ubiquitination:0.339	None
MsNAC17	119	K	Ubiquitination:0.198	None
MsNAC17	120	K	Ubiquitination:0.206	None
MsNAC17	126	K	Ubiquitination:0.217	None
MsNAC17	150	K	Ubiquitination:0.323	None
MsNAC17	163	K	Ubiquitination:0.461	None
MsNAC17	166	K	Ubiquitination:0.221	None
MsNAC17	167	K	Ubiquitination:0.271	None
MsNAC17	173	K	Ubiquitination:0.57	Ubiquitination:0.57
MsNAC17	196	K	Ubiquitination:0.45	None
MsNAC17	206	K	Ubiquitination:0.502	None
MsNAC17	218	K	Ubiquitination:0.462	None
MsNAC17	235	K	Ubiquitination:0.45	None
MsNAC17	543	K	Ubiquitination:0.226	None

Supplementary Table S2 primers

Reverse Transcription Quantitative PCR (RT-qPCR)

Gene Name	Sequences
NAC1-F	TCTGTTGAAGCAGCCTCTATGAATG
NAC1-R	CGATGGAGGTCTAACGCATATTGG
NAC2-F	ATGAGGAATGAGCGTCTTGTGTTG
NAC2-R	ACGGGCTGAGGGTGGTAAATG
NAC3-F	CGACGATATGGAGTGGTACTTCTTC
NAC3-R	TTGGACCGCCGAGCCTTG
NAC4-F	GGAAAGCCACAGGAAGAGACAAG
NAC4-R	GCACAAGAATAGAGTATGAACACTGAC
NAC5-F	CTAACTCTATTAAGCCTCTCCACATCTC
NAC5-R	CTCTCTCCATCCAAATCCTTTCAGC
NAC6-F	TGGCGACTGGCTCTTGGAAG
NAC6-R	TCAGGAGTTGGAACCGAGGAATC
NAC7-F	GTGAAGAGAATGGGAGATTACGAGTC

NAC7-R	GCTGCTGCTGATATTGTGATGATAC
NAC8-F	AGAAGGATGGGCTGTGTGTAGG
NAC8-R	TTGTGGCAGGAATGAGACTTGATC
NAC9-F	GACGATGTTCCTCAGCATTCTACC
NAC9-R	AAGAGCAAGACCACCAATGACAAG
NAC10-F	TGCTTGAGTTCCATCTTGATCCATC
NAC10-R	TTCTTCATAGTTATCTCTTGCCTCCAG
NAC11-F	GAAGACGGCGGAGGAGGAG
NAC11-R	TGAGAGTGTTGCTGGAAGAAGTTG
NAC12-F	AAGTAGTGGTATTGTTTCCTCTCAGTATG
NAC12-R	GTGGCTGGTGGTCTTGTGTAAC
NAC13-F	TGATATACCGCAGCAGCAGGAG
NAC13-R	CCAGTGATGAAGAAGCCAGAGTTG
NAC14-F	TCACTCCCAACGACCCATTCC
NAC14-R	TCCAAGCCGCAGAGAAGACTAC
NAC15-F	CATATCTTCAGTGGTTGTTGAGGTTATTG
NAC15-R	TGGTTCTTGTGAATCATTGTAGTATTGTG
NAC16-F	GACCGAACTGTGGCTTCTTCATC
NAC16-R	CATTGGTGTCATTGGTTCTTGTGTATC
NAC17-F	TCGGCTTCAGAGAATGCTTTGGTG
NAC17-R	TCTTTCTCCCTGCCCTCCTAGTTTC
NAC18-F	CCTTCATCAAGCAAGAGAATACAACTG
NAC18-R	CAATGGTGGCGTCTGTGGAAG
NAC19-F	GCAAGGTCGTGTTCGGAATCC
NAC19-R	TGGCTCCAAGGGTTTCAATTTCG
NAC20-F	CGACGATATGGAGTGGTACTTCTTC
NAC20-R	TTGGACCGCCGAGCCTTG
NAC21-F	GTGTACGAGTGTCATCCGAATCAG
NAC21-R	TGGCGAAGTAATAGGCAGCATTG
NAC22-F	CAAAGTTGCCACCAGGGTTCAG
NAC22-R	GGATTGAGTCAGATTGAGTCACCTTC
NAC23-F	GCTACTATTACTCAACAGGTGGCTTC
NAC23-R	ATCAGCGGCAGCGGACAC
NAC24-F	GAAGGTTCACACCGAAGAAGATGG
NAC24-R	TCACTGCTCCGCCGACAAG
NAC25-F	TCATCAACCAGGCTCTCACTCTC
NAC25-R	AGCAGCAGCAGTCCCTATCG
NAC26-F	GGCGGCAGAATGTTGGTGTG
NAC26-R	TGGTTGGTGGTGGTGGTGTAG
NAC27-F	GCTTCCTCGCAGTCATCTTCAG
NAC27-R	GGACGGTCGAACACTTCAACAG
NAC28-F	AGAACACCAGAAACGGCGAAAG
NAC28-R	CCAAGTAGTCCATGTCCAGTAAGC

NAC29-F	CTTCCCTTCACGAGAACAATTCAAAC
NAC29-R	CTCTGCTACATATCCTCTTGCTTGG
NAC30-F	TAACCAACTCATGGAACTTCCTCAAC
NAC30-R	TTGCTCCTGTCGTCCTCATAGTC
NAC31-F	CGATGCCAAGAAGGTTACTGACTG
NAC31-R	ACAACAACAATGCCATATCTGATTCATC
NAC32-F	CGAGTGGATGATGAATGAATACAAAGG
NAC32-R	TAACGTGGGAGCAGAAGGAGTAC
NAC33-F	AAGAGAAGAAAGTAGGGAGTATCAAAGC
NAC33-R	GGCACGGCATGGAGCATTG
NAC34-F	AACAATGCGGAGAAGGTTACTGAC
NAC34-R	GCAACAACAACAATGACATATCTGATTC
QNAC37-F	GGATGATGAGATGGTTGGAGAAGATG
NAC37-R	TTGATGCGTGGCTGAAGGTATTG
NAC38-F	CACTGACCCATTGATCCTAGACTTTG
NAC38-R	GACCACTCATCCACAAGCAACTG
NAC39-F	GTTATGGTGGTATTGTGCCGATGG
NAC39-R	TCTGTGGAGGACTCTGTGATAAAGG
NAC40-F	GATCACCCATACCATTGAGGAACTAC
NAC40-R	AATAGCGTCACTAATCATCTTGTAATTGC
NAC41-F	GCGAGGAGACCAGAGTAGTAGAATC
NAC41-R	TTGCTGCTGTGGGTAACGATTG
NAC42-F	CCGCCTTCAACAAACCAACAAAG
NAC42-R	GGGATGCCACAAGCCTATCAAG
NAC43-F	ATCCCACTCACCATTACAACAACAG
NAC43-R	CGTCCCATCGTCCCATCCTC
NAC44-F	CAACAGCGAGGAAACACAACAAAG
NAC44-R	ATATCAGCACGATGGATTGGACTTG
NAC45-F	AGATGAGAAGGAAGTGCGGTGAG
NAC45-R	TTCTTGCGGTTCTTCAACATTATTCG
NAC46-F	TCAAGAGATTATGTGGCACTTACTAGC
NAC46-R	ACAACAGATTCCTTCGTCGTCATC
NAC47-F	ATCAGGACGGCGGCAGAAC
NAC47-R	GTGGTTGTTGGTGGTGGTGTG
NAC48-F	CCCTAACTCATCATCTCCAACATCC
NAC48-R	CATCTTCCACTTCTTGCTTGAATACTAC
NAC49-F	GCTTCCGTGACCGTAAGTATGC
NAC49-R	AACACCAAAGTCTTTCTCATCCCTAC
NAC50-F	ACAATGGAACTACTAATGAAGGTGACTG
NAC50-R	CCTTGCTGATTGCCTGAGTATTCTG
NAC51-F	CCGACACCTCCTCCTCATCTG
NAC51-R	GCCTTGCTGCTCTTCAATCTCC
NAC52-F	GCGGAGGAAAGGGAGGGTTAG

NAC52-R	CGACCTGAGCATACTGATAGAGAAC
NAC53-F	TGCTTGGACGGCGACACTAC
NAC53-R	GGAGAAGAAGGAACATCACTCATCAG
NAC54-F	TCAAGAGATTATGTGGCACTTACTAGC
NAC54-R	ACAACAGATTCCTTCGTCGTCATC
NAC55-F	TTGTGTGCTGAGATGGGAGAAATTC
NAC55-R	GTGGTTGATGTGGCTGTGAAGG
NAC56-F	ACGGATCGGCTTCTACTCATCAG
NAC56-R	AATTGGTGTAGTTGTATTCTCTTGCTTG
NAC57-F	AAGGTTGCGAATGAAGGAGGAAG
NAC57-R	CTGGTTGTCTCTGTGGCTATGC
NAC58-F	AAAGGAAGAATGGGTGGTTTGTAGG
NAC58-R	GTGAGGATGTTGTTGCTTGTTGTG
NAC59-F	CGAACAAGAGGACGAAGAAGAAGAG
NAC59-R	GAATCTGCTGGGAGTGGTAATGC
NAC60-F	AGAGTTGTGTGCTGAGGGAGAG
NAC60-R	GTTGATGTGGCTGTGGTGACTG
NAC61-F	CAGCCGCCGAACTTTGGAG
NAC61-R	TCCGCCGCCAGCAACAG
NAC62-F	GATTCAGGTGCTAACTCGGAAGAC
NAC62-R	CGCCCTTTGTATCCCAGATGTTC
NAC63-F	CACTGCGACTGCTGCTGTTC
NAC63-R	AGGGAGTTTGATGGGAGGAAGATC
NAC64-F	GGTGTCTGCCTTGCCTGAAAC
NAC64-R	GTCATTGTTGTTGCCAGAGAAGTTG
NAC65-F	GATTCAGGTGCTAACTCGGAAGATG
NAC65-R	AGGTTATCAAAGAAGTATCCCAGATGTTC
NAC66-F	CAACTTGAACACGATGGACATTGAG
NAC66-R	ACCACCACTGATGAAGGACCTC
NAC67-F	CAACAATCAAATTCCACCGCCAAG
NAC67-R	GCATTCCATTAACAGACTCCCATCC
NAC68-F	CTAGAGTTGCGTGCTGAGATGGAAG
NAC68-R	GAGGAAAGGTAGTCTTGTGGTGTCTG
NAC69-F	CCTCAAGTCAGTCCTAATGCCAATC
NAC69-R	CACAGGTTGAAACATCCGTATTCTTAC
NAC70-F	ATTACATGCGAATTGCCTGAATAACG
NAC70-R	CTCAAACTCTGCCATTGGATTAACTTC
NAC71-F	GACGATATGGTGTGGTACTTCTTCAG
NAC71-R	TTGGACCGCCGAGCCTTG
NAC72-F	GAGTGATGACGAGGTTGCTGAAG
NAC72-R	ACCTATGCCAATATCCTCAAGAGAATG
NAC73-F	GAAGGTTCACACCGAAGAAGATGG
NAC73-R	TCACTGCTCCGCCGACAAG

NAC74-F	GCAGCAGTAGACCAGAAGACAAC
NAC74-R	AATAACATTAGTATCAACAGCACCAATAGG
NAC75-F	AGCGTCATCATCACCATCATCATC
NAC75-R	AGTTGCTGCTGCTGCTGTTG
NAC76-F	GCACCACACGGCAGTTCTTG
NAC76-R	CTGGCACAGGCAATAATTCATCAAC
NAC77-F	CTGGGTGGTCTGTCGCATATTTC
NAC77-R	ATATAATCCTCCAATAATCTCCGAGTCTTC
NAC78-F	GCAGCATCTTCCAGTTCGGTTC
NAC78-R	TCCATCCTGTTGTCACTTGAAGTTATG
NAC79-F	TGCCACAACCACTACCACGAG
NAC79-R	CCAGTTTCCCACTTCCGCTTTG
NAC80-F	AAGAAGTGGCAAGATGAATGAGGAG
NAC80-R	TTGAACAAGAGGAGGAAGAAGATGC
NAC81-F	GCCAGGTGATTGTGAGCAAGAG
NAC81-R	ATCCCGTCGCCTTCCAGTAAC
NAC82-F	GCTGGAGGAACGAGATAACTATGAAG
NAC82-R	AGTAAGTGCCTGAGGTTGAAAGTG
NAC85-F	AGGTGAGCCATGAAGATCCAGAG
NAC85-R	TTCCAATACCCAGTTGTTGTGAGTC
NAC86-F	TCGGCATGAAGAAGACATTGGTG
NAC86-R	AGATGATGACGCATTGGCACAC
NAC87-F	CCTCCACCTCGTTATCATTATCATCAG
NAC87-R	GCACCTCTCATCATCCCTTGTTG
NAC88-F	ACCGAGGCAACGACGAGAAG
NAC88-R	TCTTCACTCCAATCACCTTGTTATCTG
NAC89-F	ATCACATAGAAGAATGCGAAGAGTCC
NAC89-R	GTCTGGGCTGGGCGAGTC
NAC90-F	GTTTACTCTTCCAATTCCAATCACTATCG
NAC90-R	GGCTTTGTATTCATTCATCTTCCACTC
NAC91-F	GAAGAGGATTCTGACATTCTACGAAGG
NAC91-R	AAGTTCATCTGCTTGGTGGGTTTAG
NAC98-F	GTGGTGGGAAGAAGACTCATATTTCAG
NAC98-R	GCAGGCGGTGGTTGTTGTTC
NAC99-F	TGGGTTCTCTCGGAAATGAAATCG
NAC99-R	TGGGACTCGGAAGCAGGTTTG
NAC100-F	GGCGGTGGTGTTGGAGGAG
NAC100-R	CATCTGTGGTGGTGGCATTGTC
NAC101-F	CAGACCCGAGTTGCAAGAATA
NAC101-R	AGGAGTAGGAGTAGGAGTAGGA
NAC102-F	ATAAATACCCAAACAACGCCCTCTC
NAC102-R	ACAGCACAACGCAGAAGAAGAAG
NAC104-F	ACGACCACCAGCAGATCATCC

NAC104-R	GTCCCATCATCATCATCATCCTCAC
PUB30-F	GCCCTTCCTGACCCAAGTGTG
PUB30-R	TGCTGAAGTCCATCTGTAGGTGAAG
P5CS-F	ACCAAGATTCCAGTTCTCGGTC
P5CS-R	AGGAATCCTGAGCAAGACGC
RD22-F	TGATGGGGTGAATGTTAAAGC
RD22-R	GAACCCAGACAACATGATCACTG
RD29A-F	CTGAAGAAGGTAAAGGAGAAGGC
RD29A-R	CCTTCAAAATATCTCCTTGCCC
RD29B-F	CCAAATTACCATGCCTCAACG
RD29B-R	CCCTTGGACTTGTACTCTCCCA
DREB2-F	AATTCAGGATCGGAGCAAGTAT
DREB2-R	AGCTGGTACGACAAATTTGATG
DREB6.2-F	TCCCTCTTTCCCTTCTACCTC
DREB6.2-R	CTGGTGGTGCTTCTCAAATAAC
LBD1-F	ATCAACCTCATCATCACCATCAGTAC
LBD1-R	TCCTCCTCCTCCTCCTCCATTC
LBD2-F	AAGCAATGTCACCAAACTTCTAAACG
LBD2-R	GCACCAACACAGCCATAGACC
LBD3-F	CAATCTCCTCCTTCTTCTTCACCATC
LBD3-R	CACTTGTCTGCACATCTTCTCCTC
LBD4-F	AGGCACAGGCTGAGGTTGTC
LBD4-R	CCGTGAGGAATGTTGGTGATGAAG
LBD5-F	CTCCATCTTCCTCTCCTTCTTCTCC
LBD5-R	GTGGTGGTGGTGGTGGTGAG
LBD6-F	CTACAAATCCATTTCCGACCTCTCC
LBD6-R	CGCACTTCTCCGCACACTTG
LBD7-F	CTAATCCATTTCCGACCTATCCTTCAC
LBD7-R	CCAAAGACTACACTTCTCCACACAC
LBD8-F	GATGCTACGCCGAAGATGTGAC
LBD8-R	TTCTTGTCTCTTCCACCATCTGAATC
LBD9-F	AAGGTATAAGCAGCAGCAGAATCG
LBD9-R	ACCATTCCAATTACCCACCAAATCC
LBD10-F	GCAAGAACAGCAAAGAGCATTAGG
LBD10-R	TGTGGTTGACGATGATGATAAGGTG
LBD11-F	GTTGGGCAAGGACAGCAAAGAG
LBD11-R	GCGGTTGTTGGTGGTGATAAGG
LBD12-F	GAAGCAAATGCCAGGGTGAGAG
LBD12-R	CTAGAGCCAGTTGGGTTTGAAGC
LBD13-F	GAAGAAGGTGTGCTCAGGATTGTG
LBD13-R	CATCTCCTCTCTGGTGCTCTGG
LBD14-F	GATCCTGTGTACGGTTGTGTTGG
LBD14-R	AGCAGTAGTAGTGGTAATAGCGAATTG

LBD15-F	CTCTCTCCTCCAACACCAACTCC
LBD15-R	GCGGCGGCGGCTATGAG
LBD16-F	TGCCAGCCACGGACTCATAG
LBD16-R	GAAGAGGACTGTAAATGTAGAGGATGG
LBD17-F	TTATGCCGATGGTTATGCCGATG
LBD17-R	ACATTGCCTTGTTCTTCTTGTTCTTG
LBD18-F	TGCTCCTTTCTTTCCTTCAGATGAC
LBD18-R	ATTTGCTTCATACACTAAACTGCTCAC
LBD19-F	TCAATCACACTTTCTTCCTCTTCTCTC
LBD19-R	CGGCGTCGCAGCAACTTG
LBD20-F	GTAGCCTTACCAACCCAGATTGATC
LBD20-R	CTGCTGAAGTATTGATGTGGAATGC
LBD21-F	TCAACACCAACACCAACACCATC
LBD21-R	GCTGCTGAAGTATTGATGTGGAATG
LBD22-F	CGTCATCGTCATCGTCGTTTGG
LBD22-R	GGCACTCTTCAGCACATCTTCTTC
LBD23-F	GCCCTTTCTCTCCCTACTTCTCTC
LBD23-R	CAGCCACCTCCATTAGCATCTTG
LBD24-F	ATGAGGTAGGCAAGAAGTTTACAAGAG
LBD24-R	GAGGAGTTTACAGGCAGCACAAG
LBD25-F	AGGAAGTTGATGAGTTAGGCAAGAAG
LBD25-R	GAGGAGTTTACAGGCAGCACAAG
LBD26-F	TCCGAGTTATACGAGTTACATGAATCC
LBD26-R	CTCCTCTCTGCTGCTTTGAATCTC
LBD27-F	CCAAGTTATCCGAGCTACGTGAATC
LBD27-R	CTCCTCTCTGCTGCTTTGAATCTC
LBD28-F	TCACATATTCGCTCTCCAACAACAG
LBD28-R	GGTAATGAGTGCTGCTGCTGATG
LBD29-F	CGCTGCCGCCACAATCAC
LBD29-R	CCATATCTGAGGACGATGACGATG
LBD30-F	CACACCCTATTTCGGTTCAGTCC
LBD30-R	CTTGAGCCTCGTAAGTGATGGTAAC
LBD31-F	AACACCTCCGCCTCCACTAAC
LBD31-R	TCTTCCTCATCATCTTCATCATCTTGG
LBD32-F	TTGTCGTCGTGCTCTTCTTCTTG
LBD32-R	GCTTGTCCATGCTGGCTGATC
LBD33-F	AGTGTTCGGAGCAAGCAATGTG
LBD33-R	AGCCGTAAACAGGGTCTCTCAG
LBD34-F	CGCCAATAGCAACAACAATAGTGAC
LBD34-R	CCATTCATCCCACCACCACATTG
LBD35-F	CAACAAGAACAACAACAACAACAAGTG
LBD35-R	AATGCTGATGTGGATATGGATGTGG
LBD36-F	GCTCCAGCAACTACCAAACCATC

LBD36-R	CTTGACACAACCGCAAACTGAATC
LBD37-F	AAGACTCAGTGTACGGTTGTGTTAAG
LBD37-R	TCCGCCGCCAGAGTTGATG
LBD38-F	CCAGAGAGATTTGCTTGTGTTCATAAG
LBD38-R	CATGGAGTCTGCTGCTGCTTC
LBD39-F	TTTGCGAAGAAGATGCCCTCAAG
LBD39-R	TTAAATGGACTGGAAGTTGCTCTAGC
LBD40-F	GAAGGAGTTGGACGAGACTAATGC
LBD40-R	CCACGAACCCTACTACTACTAGACC
LBD41-F	CTCGTGTCCTTCATCGCAGTTG
LBD41-R	GCCACCAGAAGCATATTGATACGG
LBD42-F	CCAATTACAGCAAGCCCTAGAAGAG
LBD42-R	AGAGTAGTGCGGTGAGGAAGAAG
LBD43-F	AGCAGTCCCTATCACCTTGTATCTC
LBD43-R	CTCCTCCTTTCGCTGGCATTG
LBD44-F	AGCAGTCCCTATCACCTTGTATCTC
LBD44-R	CTCCTCCTTTCGCTGGCATTG
LBD45-F	GGGTGCTGGGAGGTGATCTG
LBD45-R	GTTGCTGGTGGTGCTGTCTG
LBD46-F	TTTACGGGTGCTGGGAGGTG
LBD46-R	TGGTGGCGGTGCTGTCTG
LBD47-F	AAGATCCTATTTATGGCTGTGTTTCCC
LBD47-R	TTAGCTGCTTGTTCCTTGACTGAAG
LBD48-F	GGTTCTGGTTCTCCTTGTGGTG
LBD48-R	GATGGTTGCGAAGTGGGTAGC
LBD49-F	TTCTGCCATGAACAAGGTGCTAC
LBD49-R	GGCGGCTTCACAACGATCAC
LBD50-F	TTCTGCCATGAACAAGGTGCTAC
LBD50-R	GCCGCTTCACAACGATCACTC
LBD51-F	GTTCGCTCCTTACTTCTGCTATGAC
LBD51-R	CGCTTCGTAAGACATGGTGATGG
LBD52-F	CGAATGCGTGATCCTGTGTATGG
LBD52-R	ATGTGTAAGTGAGAAGTTTGGAGTAGG
LBD53-F	TGGCTGCGTCGGACTCATC
LBD53-R	GGCGGCTGGAGAATGGAGAG
LBD54-F	CCTGGCTCGGTCACTGTGG
LBD54-R	GGCTGCTGCTGCTGAATCTG
LBD55-F	GGTGTGGTGTCTGGTTCATTGG
LBD55-R	CGGTTGTGGTTGGAGCATCAG
LBD56-F	CGGCAAGATCCTGAACGAACTC
LBD56-R	GAGATGAAACCAACACAACCATACAC
LBD57-F	CGGCGGCGTCATCAACTG
LBD57-R	CTTGCGTTTCGGCGAGGAG

LBD58-F	TGCCAGGCTGCCGTTGAG
LBD58-R	AGTGACGCCGCCGACATC
LBD59-F	GAGAGAGGAAGAGGGAAGGGAATG
LBD59-R	CTCGTCGTTGTCGTAGTCATCAC
LBD60-F	CCTGTTCCTCGCCAAGTTCTTC
LBD60-R	CCCACCGCTCCGTTCACC
LBD61-F	TTGGATCGGATGGTGGAGATGAG
LBD61-R	CCTTGGGCTTCGGGAGAGTC
LBD62-F	TCTCCTTCATCAACGCCGTCTC
LBD62-R	CACACCTCCCACTTCCCACTC
LBD63-F	AATGCCTACAATGGATGGAAGACC
LBD63-R	GACTGCGTTGATGAAGGAGAAGAG
LBD64-F	CCCGAGCCGAGCCACAATC
LBD64-R	TTCAGCCTCAATTCTTCTCTTCTTCAC
LBD65-F	AAGCGGACGGAGTCTGTTCAG
LBD65-R	AATCCAGCCTCAATTCTTCTCTTCTTC
LBD66-F	TACACCTCCAATCTCCTCCATTCAC
LBD66-R	AGGGTCTGATCTCGCATTCGTC
YUCCA1-F	GGGTTACCTGAATGCTTATGCT
YUCCA1-R	CACCAGTGGCAACAATCAGC
YUCCA3-F	TGGTTAGTGCTCGGAAGCAT
YUCCA3-R	GAGCTCGACTTGACCGGTTG
YUCCA4-F	AACTCCGGGGAGTATGGGAA
YUCCA4-R	AGTGCAAGACTTTGCCCTGA
YUCCA5-F	GCAAGTCCTCTTCATCCCTGT
YUCCA5-R	CCTGGAGCACACACTACGAG
YUCCA6-F	CGCTTTCCCTGTCTAAATCTTACACTT
YUCCA6-R	GAGAAGGAACGCCATTGTTGG
YUCCA7-F	GCACCCACATACATCCCCAA
YUCCA7-R	CCGTGACACGCCATTTGTTT
YUCCA8-F	AGGAAGTGCAGGTAGTCATCGTAG
YUCCA8-R	CTTAGGGATGTATGTGGGTGCATTT
YUCCA10-F	GGAGAGTTCGTGCACTCCAG
YUCCA10-R	TGTACCGGGCTACGAACAAC
YUCCA11-F	CAATCATAGTTGGAGCGGG
YUCCA11-R	AGCCTGTCGTAGGTGCGTT
YUCCA12-F	TGACTATAAGTCCGGCGTGG
YUCCA12-R	CTGAACTTCGAACCACCATCG
YUCCA13-F	ATGGGGAGGCATGCACAATG
YUCCA13-R	AGGCTCGGAATAAGCTCTGG
YUCCA14-F	TCATCGCCATCACCATCACC
YUCCA14-R	CGGTCGTAGGTCTTGAGCTG
YUCCA15-F	GCTTGCTCTATCTCAATCTTTCACT

YUCCA15-R	GGTCCTGCGCCTACAATGAT
YUCCA16-F	GGATTCCGCTGTTTGCGATG
YUCCA16-R	CGCCGGAATAATAGCCTGGT
YUCCA18-F	ACACCTACATACGTCCCCAAGAATTT
YUCCA18-R	GCGTGTTCTCGACCTTGACAT
YUCCA19-F	TCGGTACATGTCTTGCCCAG
YUCCA19-R	CCAAAGGACCTAAAGATGGCCT
YUCCA20-F	GCTACTGGGGAAGCAACTAAC
YUCCA20-R	TGGTTCGCCAAGTCTAGTGAA
MYB47-F	ACACTGAAGAGGAAGGGCACAAAC
MYB47-R	CATTTGAGCACGAGGAGACAGACC
bIP46-F	GCCAACGCACGCAATGATTCTAC
bIP46-R	TTGGGTCCTTCTTGCTCTGAACTTG
MdHistone H3-F	TGAAGAAGCCCCACAGATA
MdHistone H3-R	GTCAGTCTTGAAGTCCTGCG
MdActin-F：	GGCTGGATTTGCTGGTGATG
MdActin-R：	TGCTCACTATGCCGTGCTCA

Construction of Overexpression and Silence Vectors

Gene Name	Sequences
NAC17-pRI101-F	CACTGTTGATACATATGCCCGTCGACATGGTGACGGAGGACGATT
NAC17-pRI101-R	CCCTTGCTCACCATGGATCCGGTACCAGTGGAGAGGGATCTCCCC
PUB30-pRI101-anti-F	CACTGTTGATACATATGCCCGTCGACTGATGTAATATGGAGCCTAAAGGAT G
PUB30-pRI101-anti-R	CCCTTGCTCACCATGGATCCGGTACCGCAGCGAGTCTAGTAAACTCTACCT G

Yeast One-Hybrid (Y1H) and Yeast Two-Hybrid (Y2H)

Gene Name	Sequences
NAC17-pB42AD-F	GCCAGATTATGCCTCTCCCGAATTCATGGTGACGGAGGACGATT
NAC17-pB42AD-R	GCGAAGAAGTCCAAAGCTTCTCGAGAGTGGAGAGGGATCTCCCC
MdMYB47-pB42AD-F	GCCAGATTATGCCTCTCCCGAATTCATGGGAAGGACTCCATGTTGTG
MdMYB47-pB42AD-R	GCGAAGAAGTCCAAAGCTTCTCGAGGTCATCCGCATTGTTTTCTAAGAA
MdbZIP46-pB42AD-F	GCCAGATTATGCCTCTCCCGAATTCATGGGGTTTTCAAGAGGTTTTG
MdbZIP46-pB42AD-R	GCGAAGAAGTCCAAAGCTTCTCGAGTGACTGATCTTGTCGAGGACGA
proLBD64-laczi-F	ACCCGGGGATCTGTCGACCTCGAGCAGACCAAATCATTTTAAGATAAAA
proLBD64-laczi-R	TATACATACAGAGCACATGCCTCGAGTTTCGCTTTCTTCTTCTTCAA
proYUCCA1-laczi-F	ACCCGGGGATCTGTCGACCTCGAGGCAATAAAGGAATTAAATCAAATTCAT
proYUCCA1-laczi-R	TATACATACAGAGCACATGCCTCGAGCATATATGATCAGTGATTAGATATAAGCTT CA
proYUCCA5-laczi-F	ACCCGGGGATCTGTCGACCTCGAGCATTAATTAATTTTCCAATTCATGATCA

proYUCCA5-laczi-R	TATACATACAGAGCACATGCCTCGAGCATCTAAATGACGATATGTAACAAATTAATT
proYUCCA8-laczi-F	ACCCGGGGATCTGTCGACCTCGAGTCCACATAGATTATTAAATAATTCGAAAC
proYUCCA8-laczi-R	TATACATACAGAGCACATGCCTCGAGCATTTCTATGTTGAATTTTGGTTAGGT
proYUCCA10-laczi-F	ACCCGGGGATCTGTCGACCTCGAGATTATCATCGCGCAATATGTTGA
proYUCCA10-laczi-R	TATACATACAGAGCACATGCCTCGAGCATTTTAGGCAATGAAAATGTCTG
proYUCCA11-laczi-F	ACCCGGGGATCTGTCGACCTCGAGACATTTAATTTTAAATTCCCAAAGGT
proYUCCA11-laczi-R	TATACATACAGAGCACATGCCTCGAGCATGCTTATGATCAAAGAAGTAGTTGC
proYUCCA16-laczi-F	ACCCGGGGATCTGTCGACCTCGAGAAATCTGAAACGGTTATCTTCCCA
proYUCCA16-laczi-R	TATACATACAGAGCACATGCCTCGAGCATTTTTGACACAGCACGAGCT
proYUCCA19-laczi-F	ACCCGGGGATCTGTCGACCTCGAGGATTGTGTTTGTGTAGTTATCCTACAATC
proYUCCA19-laczi-R	TATACATACAGAGCACATGCCTCGAGCATTTGGGGTATGTGGTGAGG
ProNAC17-laczi-F	ACCCGGGGATCTGTCGACCTCGAGTGAATTCATCTTTGGCGAGAATT
proNAC17-laczi-R	TATACATACAGAGCACATGCCTCGAGCAGATAGTAGAGGACAAGCTCCTCG
poPUB30-lczi-F	ACCCGGGGATCTGTCGACCTCGAGGTAATTAAAGAATGTTTAAAATCTGCACA
poPUB30-lczi-R	TATACATACAGAGCACATGCCTCGAGGTGCTATAGATGAATAAACGGTGAAAT
PUB30-pEG202-F	TCGCAACGGCGACTGGCTGGAATTCATGGGTAACGGTGCTGCTG
PUB30-pEG202-R	TAGCTTGGCTGCAGGTCGACTCGAGATTCTTCTTTTTCGGTGCATGG

Dual-Luciferase Reporter Assays

Gene Name	Sequences
NAC17-62SK-F	GTGGATCCCCCGGGCTGCAGGAATTCATGGTGACGGAGGACGATT
NAC17-62SK-R	GAATTGGTACCGGGCCCCCCCTCGAGAGTGGAGAGGGATCTCCCC
PUB30-62SK-F	GTGGATCCCCCGGGCTGCAGGAATTCATGGGTAACGGTGCTGCTG
PUB30-62SK-R	GAATTGGTACCGGGCCCCCCCTCGAGATTCTTCTTTTTCGGTGCATGG
bZIP46-62SK-F	GTGGATCCCCCGGGCTGCAGGAATTCATGGGGTTTTCAAGAGGTTTTG
bZIP46-62SK-R	GAATTGGTACCGGGCCCCCCCTCGAGTGACTGATCTTGTCGAGGACGA
proLBD64-LUC-F	ACTCACTATAGGGCGAATTGGGTACCCAGACCAAATCATTTTAAGATAAAA
proLBD64-LUC-R	AGCTTATCGATACCGTCGACCTCGAGTTTCGCTTTCTTCTTCTTCAA
proYUCCA11-LUC-F	ACTCACTATAGGGCGAATTGGGTACCACATTTAATTTTAAATTCCCAAAGGT
proYUCCA11-LUC-R	AGCTTATCGATACCGTCGACCTCGAGCATGCTTATGATCAAAGAAGTAGTTGC
ProPUB30-LUC-F	ACTCACTATAGGGCGAATTGGGTACCGTAATTAAAGAATGTTTAAAATCTGCACA
ProPUB30-LUC-R	AGCTTATCGATACCGTCGACCTCGAGGTGCTATAGATGAATAAACGGTGAAAT
PUB30-NLUC-F	AACACGGGGGACGAGCTCGGTACCCATGGGTAACGGTGCTGCTG
PUB30-NLUC-R	CGGGACGCGTACGAGATCTGGTCGACCTCGAGATTCTTCTTTTTCGGTGCATGG
NAC17-CLUC-F	TCTCGTACGCGTCCCGGGGCGGTACCATGGTGACGGAGGACGATT
NAC17-CLUC-R	TACGAACGAAAGCTCTGCAGGTCGACTCAAGTGGAGAGGGATCTCC

Electrophoretic Mobility Shift Assay (EMSA) and Protein Related Experiments

Gene Name	Sequences

NAC17-GEX-F	TCCGCGTGGATCCCCGGAATTCATGGTGACGGAGGACGATT
NAC17-GEX-R	GATGCGGCCGCTCGAGTCGACCAGTGGAGAGGGATCTCCCC
NAC17-GFP-F	CACTGTTGATACATATGCCCGTCGACATGGTGACGGAGGACGATT
NAC17-GFP-R	CCCTTGCTCACCATGGATCCGGTACCAGTGGAGAGGGATCTCCCC
MYB47-GEX-F	TCCGCGTGGATCCCCGGAATTCATGGGAAGGACTCCATGTTGTG
MYB47-GEX-R	GATGCGGCCGCTCGAGTCGACCGTCATCCGCATTGTTTTCTAAGAA
BZIP46-GEX-F	TCCGCGTGGATCCCCGGAATTCATGGGGTTTTCAAGAGGTTTTG
BZIP46-GEX-R	GATGCGGCCGCTCGAGTCGACCTGACTGATCTTGTCGAGGACGA
proLBD64-Cold-F	TTGGCGAATAGGTCACGTATTTTGTAGGGC
proLBD64-R	GCCCTACAAAATACGTGACCTATTCGCCAA
proLBD64-Mutation-F	TTGGCGAATAGGTTTTTTATTTTGTAGGGC
proLBD64-Mutation-R	GCCCTACAAAATAAAAAACCTATTCGCCAA
proYUCCA11-Biotin-F	TAGGCACCAACTACACGATATTTTTTCTAT
proYUCCA11-Cold-F	TAGGCACCAACTACACGATATTTTTTCTAT
proYUCCA11-R	ATAGAAAAAATATCGTGTAGTTGGTGCCTA
proYUCCA11-Mutation-F	TAGGCACCAACTATTTTTTATTTTTTCTAT
proYUCCA11-Mutation-R	ATAGAAAAAATAAAAAATAGTTGGTGCCTA
proMdNAC17-Biotin-F	CCAGACAAATTAACGAGGGTAATACATTGC
proMsNAC17-Biotin-F	CCAGACAAATTAACAAGGGTAATACATTGC
proMsNAC17-Cold-F	CCAGACAAATTAACAAGGGTAATACATTGC
proMdNAC17-R	GCAATGTATTACCCTCGTTAATTTGTCTGG
proMsNAC17-R	GCAATGTATTACCCTTGTTAATTTGTCTGG
proMdPUB30-Biotin-F	ATTCTTCAATAAGCACGTGAAGAGATAGAA
proMsPUB30-Biotin-F	ATTCTTCAATAAGCGCGTGAAGAGATAGAA
proMsPUB30-Cold-F	ATTCTTCAATAAGCGCGTGAAGAGATAGAA
proMdPUB30-R	TTCTATCTCTTCACGTGCTTATTGAAGAAT
proMsPUB30-R	TTCTATCTCTTCACGCGCTTATTGAAGAAT

Promoter Activity Analysis

Gene Name	Sequences
NAC17pro-1301-F	ACAGCTATGACCATGATTACGAATTCTGAATTCATCTTTGGCGAGAATT
NAC17pro-1301-R	TTACCCTCAGATCTACCATGGCAGATAGTAGAGGACAAGCTCCTCG
NAC17p1-1301-F	ACAGCTATGACCATGATTACGAATTCGATAAAATGAGCTTGGAGTGGAGT
NAC17p1-1301-R	TTACCCTCAGATCTACCATGGTCACCTCATGATTGACTTTTAAGACA
NAC17p2-1301-F	ACAGCTATGACCATGATTACGAATTCAAACTTCTCATCCCCTCTCTCG
NAC17p2-1301-R	TTACCCTCAGATCTACCATGGCCTACATAAACACAGTAGGACTCTCCTA
PUB30pro-1301-F	ACAGCTATGACCATGATTACGAATTCATTGGTGTTTTTATATGTCCTTCGTC
PUB30pro-1301-R	TTACCCTCAGATCTACCATGGACCGTTACCCATTTGAGAGAAATTA
PUB30p1-1301-F	ACAGCTATGACCATGATTACGAATTCAATTATTTAATTACATTTGTTATCGTACTG
PUB30p1-1301-R	TTACCCTCAGATCTACCATGGATTTCGACAAACTAGTGTAATAAAATTTTTAT
PUB30p2-1301-F	ACAGCTATGACCATGATTACGAATTCTGTTTCTTTGGTTTGTGCGGT
PUB30p2-1301-R	TTACCCTCAGATCTACCATGGCTCCTCAGTCTTTAGCCAGACAGA

200
