# Supplementary figures and images for: The NAC17–PUB30 module enhances drought stress tolerance by regulating adventitious root development in apple
Source: Plant Physiol. 2026 Jul 10;201(3):kiag488. doi: 10.1093/plphys/kiag488 (PMC13418357; doi:10.1093/plphys/kiag488)

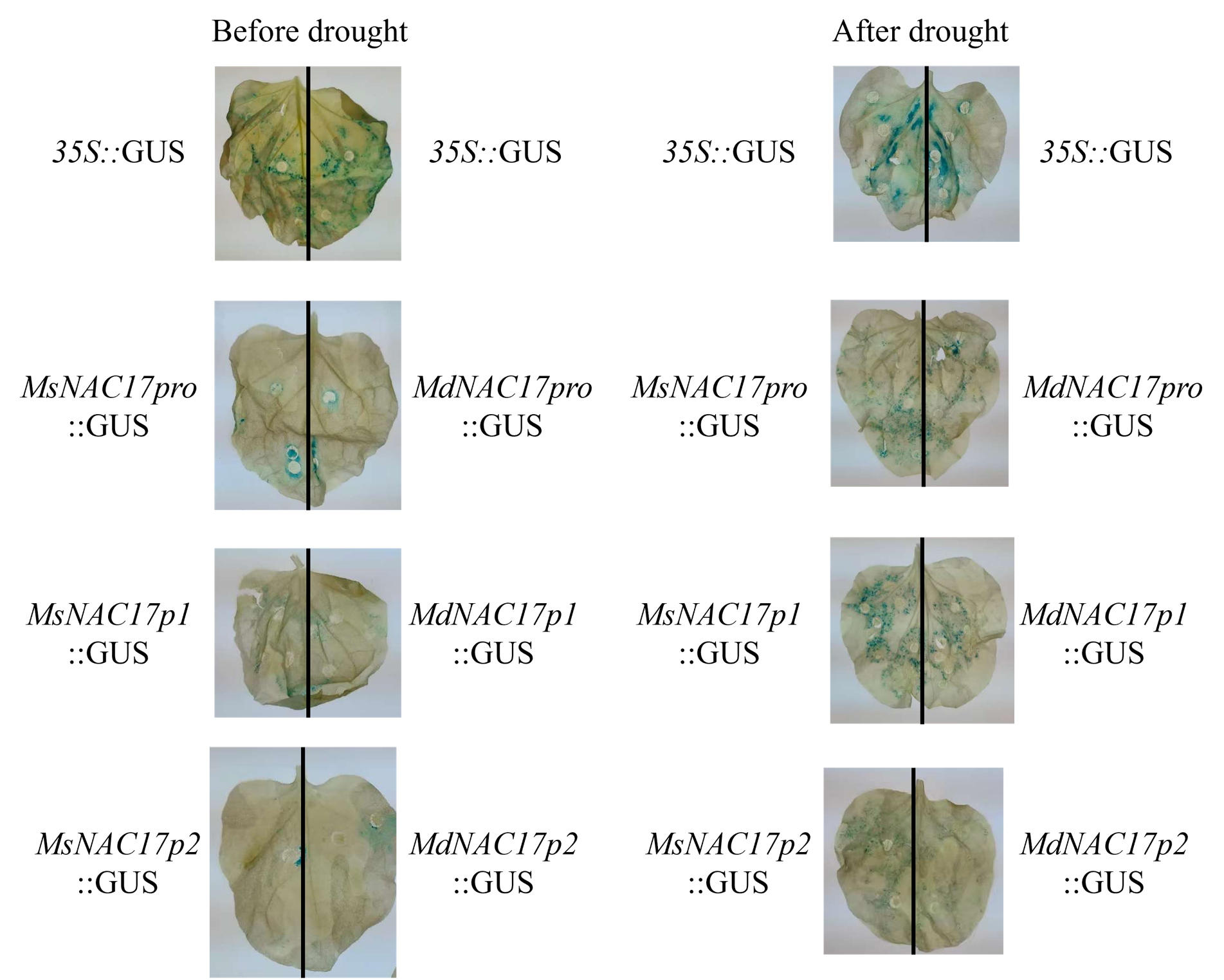

Supplement: kiag488_Supplementary_Data [file kiag488_supplementary_data.zip › Fig. S13B.tif]
